# Diversity of Effects of Mechanical Influences on Living Systems and Aqueous Solutions

**DOI:** 10.3390/ijms26125556

**Published:** 2025-06-10

**Authors:** Sergey V. Gudkov, Vladimir I. Pustovoy, Ruslan M. Sarimov, Dmitriy A. Serov, Alexander V. Simakin, Ivan A. Shcherbakov

**Affiliations:** Prokhorov General Physics Institute of the Russian Academy of Sciences, Vavilove St. 38, 119991 Moscow, Russia; s_makariy@rambler.ru (S.V.G.); pustovoy@nsc.gpi.ru (V.I.P.); rusa@kapella.gpi.ru (R.M.S.); dmitriy_serov_91@mail.ru (D.A.S.); avsimakin@gmail.com (A.V.S.)

**Keywords:** mechanobiology, mechanical action, ultrasound, vibration, water solutions, cell differentiation, ROS

## Abstract

Water is the basis of life. Any factors acting on water will also affect the functioning of living organisms, including humans. Mechanical effects are as ubiquitous as temperature or magnetic fields. Numerous works have been devoted to the action of mechanical impacts on living systems, aqueous solutions, and water. However, no unified theory that would allow predicting the consequences of mechanical effects on living organisms based on their characteristics. In this review, we have attempted to systematize the available quantitative data on the effects of mechanical impacts on living organisms, cells, aqueous solutions, and purified water. In addition, in this review, we provide a basic overview of the variety of mechanical effects and the mechanisms of their realization. The responses of living systems and aqueous solutions depend quantitatively on different sets of characteristics of the vibration action. The magnitude of responses of living systems (cells and organisms) to mechanical action correlates with frequency, acceleration, and force. Mechanical action changes the characteristics of water and aqueous solutions as a function of frequency, acceleration, and duration. The data obtained may find application in a wide range of fields: from analytical chemistry and pharmacology to environmental protection.

## 1. Introduction

Water and aqueous solutions are subjected to mechanical action on a daily basis: from bars to pharmaceutical production, from water purification systems to the propellers of supertankers, from air humidifiers to watering plants in agriculture [1,2,3,4]. Mechanical action on aqueous solutions is used for various tasks in human activities. The most common application of mechanical action to aqueous solutions is to dissolve and/or mix and/or change temperature and/or transfer torque/force and/or wet something [5]. Mechanical effects are even used in nanotechnology, e.g., to concentrate particle separation molecules from the solvent by flotation [6] or to control nanoparticle aggregation [7]. More than a hundred years ago, it was found that mechanical impacts of high intensities are observed to alter/damage organic molecules in water. For example, denaturation of proteins in aqueous colloids is observed when they are mechanically impacted [8]. This discovery was later proposed to be used as a stress test to check the quality of antibodies [9]. Today, a whole section of chemistry (mechanochemistry) studies changes in the properties of substances and their mixtures, as well as physicochemical transformations under mechanical effects [10]. The negative effects of prolonged mechanical impact on the level of living tissues and organisms have been well described. There is a separate group of so-called vibration diseases, which belong to occupational diseases and affect various body systems. The complex of vibration diseases affects both pathologies of the nervous system (peripheral neuropathy) and musculoskeletal system (aseptic necrosis, fatigue fractures, degenerative joint disease, carpal tunnel syndrome), as well as the cardiovascular system (Raynaud’s syndrome) [11]. Even though modern working conditions are designed to provide a high level of safety in chronic vibration exposure due to modernization of impact tools and new technological elements of vibration protection [12], the incidence of vibration diseases in workers of construction, mining, metalworking industries and operators of heavy machinery is still about 10–40%, depending on the length of service and occupation [13]. It is known that the highest frequency of vibration disease is observed among workers in the mining industry and construction workers. The risk of vibration diseases increases significantly after 5–7 years of work in these industries. Unfortunately, there are no unified working recommendations on the prevention and ideal treatment of vibration diseases. Unfortunately, there are no unified working recommendations on prevention and ideal therapy of vibration diseases today [14].

In order to create more effective ways to treat aqueous solutions, to create new biotechnological approaches, to improve the quality and safety of life, it is necessary to understand in detail the regularities of mechanical (especially weak) effects on water, aqueous solutions and living organisms, which are 70–90% composed of water [15].

Almost 300,000 works have been published on the mechanobiology issues (https://pubmed.ncbi.nlm.nih.gov/?term=mechanobiology accessed on 26 March 2025). However, there is no common unified theory linking the characteristics of heterogeneous mechanical effects (vibration, shear stress, pressure, and sound action) to date. In this review, we have attempted to find general regularities that determine the magnitude of the effect of mechanical action on aqueous solutions and biological objects depending on the characteristics of mechanical action (duration, frequency, acceleration, force, and other features).

In addition, this review will describe the main effects of mechanical action on purified water, aqueous solutions of organic compounds, cell cultures, and whole organisms. The main mechanisms of the realization of mechanical impact effects will be discussed.

## 2. Diversity of Effects of Mechanical Influences on Living Systems and Aqueous Solutions

### 2.1. Impact Types

Mechanical effects on aqueous solutions are ubiquitous around us in all spheres of life: household, technology, agriculture, and the pharmacological industry [16,17,18,19]. However, the exact dependence of the effects of mechanical actions on water and aqueous solutions on their characteristics has not yet been determined. The mechanical effects that can be exerted on water, aqueous solutions, and living organisms are quite diverse. They include the following: sinusoidal vibrations [20,21], different mixing modes (turbulent, laminar, etc.) [22,23,24], centrifugation [25], shear stress in constant flow [26], dropping from a height in the form of drops or vials [24,27,28], formation of nanobubbles under pressure, and others [29]. Separately, ultrasound exposure is the subject of research as a potential therapeutic agent [30,31]. In addition, mechanical effects are often used simultaneously with other weak effects, such as weak permanent and alternating magnetic fields, electric fields, and others [32,33,34,35]. Vibration effects have an impact on the physicochemical properties of solutions. Their effects have already found application in practice, in particular, it is used in the technology of preparing vibratory iterations [16,36] and the technology of preparing highly dilute solutions [37,38].

In the present work, we attempted to systematize the available literature data and to identify the general regularities that determine the magnitude of mechanical effects on water, aqueous solutions, and living organisms based on the characteristics of these effects.

Analyzed works can be conditionally divided into works on the study of mechanical effects such as shaking, impact, ultrasonic effects, etc. (Appendix A). In order to evaluate quantitative dependencies of the magnitude of the effects of impacts, we further divided them into two groups: mechanical (vibration) and ultrasonic.

### 2.2. Effects of Mechanical Influences

According to the objects of study, the analyzed works can be divided into three large groups:

1. The whole organism in vivo;
2. Cell cultures;
3. Aqueous solutions or deionized water.

For this reason, we will use this classification when describing the effects.

#### 2.2.1. Mechanical Impact on Living Systems

Most of the work on in vivo mechanical effects focuses on the cardiovascular, immune, and musculoskeletal systems. The circulatory system can be a target of weak mechanical effects. At the systemic level, whole-body vibration causes an increase in HR and blood pressure [39]. In rodents, it was shown that sinusoidal mechanical vibration reduced the number of capillaries (lectin-positive vessels), venules, and arterioles (α-actin-positive blood vessels) in muscle fibers compared to controls [20]. Another work showed a decrease in vascular lumen under the effect of vibration by increasing the smooth muscle work of microvessels [40]. Micro-damage of endothelial cells (increase in the number of vacuoles, membrane ruptures), as well as damage to the integrity of the inner walls of blood vessels, were found at the cellular-tissue level [41]. Intermittent mechanical impacts, even on isolated body parts, can induce a proinflammatory response in the body, expressed in the activation of NFAT-dependent signaling pathways, increased IL-6 synthesis, and oxidative stress [40,41]. Intermittent jade vibration of isolated limbs increases the activity of total plasma creatine phosphokinase (t-CPK), the excess of which is a marker of tissue damage [42,43]. However, more moderate loads with low acceleration and high total duration can have a positive effect on bone mineralization in humans by reducing TRAP-dependent osteoclast activity [44,45].

In humans, whole-body vibration after 8 days increased plasma concentrations of the osteoporosis markers P1NP and CTx [46,47]. An increase in leg skin temperature has also been reported after vibration impact [46]. In another study on rats with hip implants, an improvement in bone-implant contact was shown [48]. In a rat osteoporosis model, whole-body vibration promoted protection against obesity and increased bone mineralization, and worsened their mechanical characteristics (stiffness, maximum load in vertebra compression test) [28]. The mechanism of the vibration effect involves a decrease in the viability of osteoblasts, a reduction in alkaline phosphatase (ALP) activity, and a decrease in the expression of osteogenesis regulators, including MMP-2 and OSX [28,49,50]. A positive effect of intermittent vibration on muscle strength assessed by apparent mass and mechanical resistance was observed in studies on volunteers [51].

A positive effect of mechanical vibration on human respiratory system functions (peak inspiratory flow rate, inspired volume, and expired volume) has been described [52].

Weak mechanical vibration can also act on the nervous system, in particular, it increases the diameter of myelin fibers in muscles in rabbits [53]. Similar results were obtained in rats, demonstrating an acceleration of regeneration of the injured nerve due to an increase in IGF-I expression [54]. In part of the in vivo studies, the quantitative acceleration-effect or frequency-effect relationship was bell-shaped or reached a plateau after certain values [48].

Shaking can also act on plants in addition to animals. In particular, it has been shown that shaking at an angle of 45° slows seed germination by reducing seedling stem length, number of leaves, basal diameter, and total shoot biomass [55].

The effects of mechanical action (centrifugation, shear stress, vibration) depend largely on the cell lineage and may be opposite for different cell types. Periodic application of centrifugal force (100–200 g centrifugation) to culture stem cells from human exfoliated deciduous teeth inhibited their proliferation [25]. Vibrations altered the proliferation rate of Chinese hamster ovary cells and T cells [56]. The direction of the effect depended on the frequency: at 30 Hz, an increase in proliferation was observed, and at >50 Hz, an inhibition was observed [56]. Sinusoidal vibration causes a dramatic decrease in the viability of fibroblasts [55]. Erythrocytes become more susceptible to lysis under shock shaking [57]. Vibration caused an acceleration of proliferation and differentiation in MC3T3-E1 osteoblast culture [26]. The degree of differentiation was assessed by alkaline phosphatase activity, osteocalcin levels, *Runx2*, and *Osx* gene expression [26,58]. Notably, the application of constant shear stress (ductal culturing) did not produce similar results as vibration [26]. Cyclic shear stress (agitation of the medium) also enhanced the differentiation of primary fibrochondrocytes: a number of BrdU-positive cells, ACAN, and SOX9 expression [59]. Vibration increased the proliferation of embryonic cell line GD25 cells and human osteoblast-like cells [60,61]. Vibration also decreased alkaline phosphatase activity in the primary culture of human osteoblast-like cells [60]. Shear stress increases the metabolism of human periodontal ligament cells (hPDLSC), increases the expression and activity of indoleamine 2,3-dioxygenase, cyclooxygenase-2, kynurenine synthesis, TGF-β1 growth factor expression, and decreases the synthesis of the pro-inflammatory cytokine IFN-β [62]. The combination of these latter effects suggests an anti-inflammatory effect of the applied exposure [63]. Vibration stimulated the synthesis of osteoarthritis markers collagen II and aggrecan in pig chondrocytes [64,65]. On the other hand, vibration increased proliferation and protected the MLO-Y4 osteocyte lineage from apoptosis [66]. The molecular mechanisms of the observed effects are decreased expression of RANKL and TNF-α [66].

However, constant shear exposure causes an increase in collagen content in mesenchymal stem cells MSCs [67]. The attenuation of differentiation in pluripotent iPSC stem cells under the influence of vibration has been described [68]. For another type of stem cell (human adipose-derived stem cells, hASCs), an increase in A cytochrome P450 monooxygenase CYP1B1 expression and metabolic activity has been described [69]. The combination of mechanical shaking and weak alternating magnetic fields causes an enhancement of fMLF- and PMA-induced ROS production by mouse granulocytes or ‘neutrophil respiratory burst’ [32,33]. Continuous shear stress can affect the calcium homeostasis of endotheliocytes and induce periodical changes in resting calcium concentration, and alter the characteristics of calcium oscillations on the background of AChR ligand application [70,71].

The application of constant pressure can be considered as a special case of mechanical action [72]. For example, ultrasonic influence can be expressed not only in intensity and frequency but also in the pressure exerted by the ultrasonic wave on the sample [73]. The application of constant pressure of different magnitudes, in the case of cells, can be modeled using substrates with different densities. Growing differentiated dendritic cells on substrates of different densities can alter cell metabolism by reducing the efficiency of glycolysis, lactase activity, and oxygen consumption [72]. In addition, a softer substrate (calculated pressure of 2000 Pa) increased oxygen consumption and gene expression of glycolysis enzymes Hexokinase II (Hk2) and glucose transporter GLUT1 (Slc2a1) compared to the harder standard substrate (calculated pressure of 50,000 Pa) [72]. In addition, cells grown on the harder substrate showed more pronounced immune activity: their immune memory and antitumor activity were improved when injected in vivo into mice with solid tumors [72]. Thus, it can be concluded that the density of the extracellular matrix is one of the potential mechanisms of regulation of immune cell functioning. Water-swelling polymers such as poly(ethylene glycol) (PEG), poly(vinyl alcohol) (PVS), poly(2-hydroxyethyl methacrylate) (PHEMA), and poly(acrylamide) (PAAM) can form elastic hydrogels that can reproduce the basic mechanical characteristics of soft tissues and be used for mechano-mediated regulation of cell function in culture [74,75,76].

The wastewater treatment problem has been relevant since ancient times and is still unsolved. The search for new, cheaper, faster, and more effective methods of wastewater treatment continues [77,78]. Vibration can be applied to increase the efficiency of bioelectrolytic wastewater treatment systems. In particular, constant agitation with nitrogen purging of water has been shown to increase nitrate uptake and sodium removal from wastewater by *T. denitrificans* bacteria immobilized on the electrode. At the same time, the voltage on the electrodes increased almost 2-fold compared to non-stirred water [79].

A summary of the mechanical impact effects is shown in Table 1.

#### 2.2.2. Mechanical Impact on Water Solution

Mechanical effects on aqueous solutions include shaking, tumbling, and high-pressure blowing (formation of microbubbles). Mechanical shaking of pure water at 30 Hz also changed the pH to 0.6 pH units [80]. The effect may seem small, but it must be recalled that physiological pH in a living organism is actively maintained within narrow limits. For example, a deviation of blood pH by a fraction of a unit from the normal range of 7.35–7.45 can lead to dangerous conditions such as acidosis or alkalosis [81].

Water stirring also enhances the absorption of water in the terahertz region 140–230 cm^−1^ [82] and changes the characteristics of water fluorescence in the UV region: the absorption maximum shifts from 250 nm to 340 nm and the emission maximum from 270 to 330 nm [83]. The fluorescence intensity increases ~5 times [83]. Prolonged (several hours) shaking changes the spectral parameters of water in the UV region. Fluorescence increases at a wavelength of 255 nm, and light scattering increases at a wavelength of 355 nm [83].

Vertical mechanical shaking or agitation causes the generation of hydrogen peroxide H_2_O_2_, singlet oxygen, and OH-radicals in pure water [80,84,85]. It is noteworthy that the amount of ROS generated is positively dependent on the frequency, amplitude, and duration of vibration impact [86]. The type of agitation affects the efficiency of ROS generation: turbulent agitation causes the generation of ~10 times more ROS than laminar agitation [85]. However, it is worth noting that under the conditions studied by the authors (frequency 10–30 Hz, amplitude 1 cm), the concentration of ROS is very small (tens of nM), which is less than the described concentrations exerting biological effects [87,88]. The falling of deionized water drops on a solid surface also causes the generation of H_2_O_2_, and OH-radicals in water, the concentration of which grows in proportion to the height from which the drop fell [27]. High-frequency mechanical vibration of water coated with a layer of oil increased its redox potential by 2.5 times compared to untreated samples [89].

Mechanical impact changes not only the properties of water, but aqueous solutions too (both one-substance solutions and complex mixtures). Circular agitation of an aqueous solution of interferon-gamma (IFNγ) increases its electrical conductivity compared to an unagitated solution [34]. Vertical shaking at frequencies of 10–50 Hz of deionized water causes an increase in its spontaneous chemiluminescence for at least the next 15 min [21]. The addition of 1 mg/mL of BSA protein to the water cancels the effects of mechanical action on the water [21]. Circular agitation of an aqueous solution of polyclonal antibody to IFNγ enhances the luminescence of the solution [16]. A series of impacts also causes luminescence enhancement of aqueous solutions of carbohydrates, using lactose as an example [17]. Vertical shaking of an aqueous solution of human immunoglobulin causes the generation of ~200 nm nanobubbles [23,90]. At the same time, the concentration of molecular oxygen in water and water temperature (by a fraction of a percent) increased [80]. The magnitudes of the effects were proportional to the frequency of shaking.

Dropping of vials with aqueous buffer solutions of anti-antistreptavidin IgG1 enhances protein aggregation as assessed by the number of suspended particles and separation of proteins from the walls after treatment with urea and guanidine hydrochloride [24,91]. Shaking even with small amplitudes (less than 0.1 mm) and frequencies (10 Hz) retarded protein crystallization using chicken egg lysozyme in a buffer solution, for example [92]. The change in flow rate affects the precipitation of lysozyme after treatment with a high concentration of NaCl [93], indicating possible conformational changes of the protein molecule, and could potentially be applied in protein production technology.

The method of solution stirring can change the geometric characteristics of crystals formed during the evaporation of aqueous extracts of medicinal plants [22]. Turbulent mixing produces less ordered and more heterogeneous crystals, while circular mixing produces more ordered structures (having less lacunarity and entropy) with more pronounced fractal properties [22]. Serial impacts alter the shape of crystals formed from plant and inorganic components during the evaporation of aqueous extracts of echinacea, baptisia, and luffa [23].

Microfluidic agitation can also be considered a variant of mechanical action. It has been shown that microfluidic mixing of a solution of acetate of europium (AcEu) causes a change in its Raman spectra in the region of 1360–1770 cm^−1^, in particular increasing the intensity of the OH groups’ stretching vibrations peak [94]. The stretching of a column of an aqueous solution of hydrogel precursors between two sites affects the spatial structure of the synthesized hydrogel. By varying the tension alone, a controlled (including mathematically predicted) change in the net structure of the final polymer can be achieved [95].

Passing deionized water through a nozzle at high speed causes the creation of water nanodroplets that contain hydrogen peroxide. The size of the microdroplets determined the concentration of hydrogen peroxide that was generated in them. The smaller the microdroplet, the greater the concentration of hydrogen peroxide. It is noteworthy that degassing water by nitrogen purging did not cause a significant decrease in hydrogen peroxide generation in the microdroplets compared to the control. Hence, the process may occur without the involvement of oxygen [90]. The creation of microdroplets can be one of the ways to catalyze chemical reactions. In particular, when microdroplets of aqueous solution of substrates of the Pomeranz-Fritsch reaction are squeezed together under accelerated nitrogen purging, the reaction rate can be increased by 5–7 times compared to the reaction rate in the control solution [29]. The combination of mechanical and electrical effects can weakly change the volt-ampere characteristics of solutions of organic and inorganic mercury compounds [35]. Stirring of the solution can also be attributed to mechanical effects. Stirring of water creates nanobubbles in it, the number of which is proportional to the duration of stirring [96].

### 2.3. Ultrasound Impact

Works on the ultrasound (US) impact on living systems are usually represented by studies on cell lines and aqueous solutions [95]. The influence of ultrasound on living organisms is investigated by the new field of sonobiology [96]. We will combine in vivo and in vitro results in one section when discussing the effects.

#### 2.3.1. US Impact on Living Systems

Periodic US exposure promoted more effective tissue regeneration in rats with a model of muscle injury (transection), which was estimated by total protein mass and muscle fiber diameter due to increased myocyte proliferation [97,98]. The US may also accelerate drug delivery in eye tissues [99]. It was demonstrated that exposure to ultrasound at a frequency of 1 MHz and an intensity greater than 8 W/cm^2^ disrupted the ER structure. Immediately after exposure, disruption to the ER microstructure was observed in all the cells studied, and after three and 24 h, recovery of the ER was observed in 16% and 26% of cells, respectively [100]. The effect of ultrasound depends on the organism’s taxonomic classification. For example, ultrasound did not affect *Vicia faba* root meristem cells at a frequency of 1.1 MHz and 8 mW/cm^2^ [101], whereas animal cells showed effects at lower frequencies and intensities.

The works on the influence of the US on cell proliferation and differentiation are quite numerous. There are data on the effect on the primary culture of human fibroblasts, human amnion-derived mesenchymal stem cells (hAD-MSC), and murine myoblasts C2C12 [102,103,104]. Ultrasound causes the acceleration of human fibroblasts’ proliferation, increased expression of collagen, and secretion of proinflammatory cytokines IL-1β and IL-8 [102]. The regulation of proliferation occurs with the participation of EGFb regulatory factors [102,105]. Ultrasound enhances the secretion of angiogenesis stimulator VEGF by fibroblasts, which may indicate the ability of the US to enhance angiogenesis in tissues [102,106]. The effect is slightly dependent on the intensity and, to a lesser extent, on the US frequency [102]. Ultrasound enhances the differentiation and division rate of hAD-MSC stem cells by increasing protein synthesis of the cell cycle regulators Cyclin D1/β, Cyclin B1/β, Cyclin E1/β, and Cyclin A1/β [103]. Since the concentrations of all four forms of Cyclins were increased, it can be concluded that US affects beyond the G1, G2, and M stages of the cell cycle [107]. In terms of blood cells, the following were recorded in the US: induction of Ca^2+^ influx and platelet aggregation; induction of apoptosis in the HL-60 and U937 leukocyte cell lines; hemolysis of erythrocytes; and an increase in the number of chromosomal aberrations in whole blood cells [108,109,110,111]. Ultrasound with a frequency of over 5 MHz can alter the spatial orientation of platelets [112].

Ultrasound almost doubled the rate of division of mouse myoblast culture and bovine aortic endothelial cells, and promoted differentiation, expressed as an increase in the length of myotubes and the number of cells fused into one myotube, and regeneration of myofibrils [104,113,114]. At the molecular level, the expression of myogenin, Pax7, COX2, and the inhibition of pro-inflammatory reactions were observed [113,115]. The action of the US on neuronal cells accelerates the differentiation, assessed by the expression of markers Alk, Cenpf, and Pcdh17, as well as the expression of actin involved in the formation of outgrowths [116].

The US destroys bacterial spores and cells, and induces DNA release from them [117,118]. The US also accelerates DNA plasmid transfer in bacterial cultures [119]. The US inhibits plant photosynthesis [120]. A summary of US impacts on living systems is shown in Table 2.

#### 2.3.2. US Impact on Water Solutions

The ultrasonic treatment of deionized and artesian water changes its pH by 0.3–0.6 and 0.5–1.05 pH units, respectively [121]. At the same time, the US treatment causes acidification of deionized water and alkalization of artesian water, indicating the role of dissolved salts in the effect of ultrasonic treatment on water pH [121]. US treatment promotes accelerated crystallization of HEWL in acetate buffer [93]. Currently, approaches are being developed for the practical application of the US in accelerating the precipitation of soya proteins in biotechnological processes [122,123]. In addition to proteins, ultrasonics accelerate the precipitation of clay particles in aqueous colloids [124]. Increasing the duration of the US treatment favors faster precipitation. Methods for accelerating precipitation using ultrasonics have also found applications in other fields, such as nanoparticle synthesis [4,125]. The US can catalyze H_2_O_2_ generation reactions in water in the presence of solid catalysts (Bi_3_TiNbO_9_) [126]. Currently, there is a problem of finding a cheaper and safer method of hydrogen peroxide synthesis than the currently used anthraquinone process [127], so the use of US and solid catalysts for this purpose seems reasonable.

## 3. Mechanisms of the Mechanical Impact Effects

### 3.1. Receptor Response of Living Systems. Mechanotransduction

Mechanotransduction is a group of processes of signal conversion from receptor structures of cells into molecular and biochemical responses of cells [128,129,130]. Mechanotransduction involves millisecond processes of the response of sensitive molecules and structures (usually receptors) and triggering of signaling pathways (changes in transcriptional activity, auto- and paracrine regulation), which further determine the fate of cells and the organism as a whole [31]. Mechanotransduction events can be conditionally divided into three main stages: mechanosensing, mechanotransmission, and mechanoresponse. Classically, these processes appear to be sequential; however, parallel and cyclic processes are possible [131,132,133].

The first stage includes receptor perception of a mechanical stimulus due to changes in the conformation of receptor proteins. Mechanosensing can occur passively in either an outside-in or an inside-out way. Outside-in scheme realized through events: “external force → change of protein structure → signal transduction”.

When implementing the inside-out mechanism, two modes are possible:

1. “elements of cytoskeleton → creation of internal force on the mechanoreceptor from the intracellular matrix → activation of mechanoreceptor → detection of changes in the extracellular matrix under external mechanical stimulation” (inside out).
2. “absence of internal force on the mechanoreceptor from the intracellular matrix → inhibition of mechanoreceptor → inhibition of detection of changes in the extracellular matrix under external mechanical stimulation”

The advantage of the first pathway is the ability of the cell to respond to mechanical stimuli at any time (excluding refractoriness). The advantage of the second method is the possibility for selective “on/off” mechanosensitivity by the cell [128].

Examples of outside-in receptors are TRP receptors (TRPV1, TRPV2, TRPV4, etc.), which can open directly upon mechanical membrane deformation or open indirectly via a phospholipase A2 (PLA2)-dependent pathway [134,135]. Nuclear proteins associated with cytoskeleton proteins transmit mechanical stress to the nuclear apparatus and alter the transcriptional activity of the cell. For example, LINC (Linker of Nucleoskeleton and Cytoskeleton) complexes include a group of protein INM on the inner nuclear membrane, SUN1 and SUN2 proteins in the nuclear envelope lumen and outer nuclear membrane, and KASH proteins on the outer side of the outer nuclear membrane [136]. KASH protein mediates interactions with actin cables through Nesprin 2. A-type and B-type lamins are also involved in mechanoreception through the spatial reorganization of chromosomes [137,138,139]. Some subtypes of K^+^ channels, K2P and ASIC families, can also respond to mechanical stress [140,141,142,143]. Different ASIC subtypes can act as primary mechanosensing receptors (ASIC1a, ASIC1b) or modulate and regulate the pain pathway and nociception as a special case of mechanoreception (ASIC2 and ASIC3) [14,144,145,146].

The inside-out system consists of actin, talin, myosin II, vinculin, and integrin proteins bound in the extracellular matrix from the fibrillar proteins surrounding the cell [147,148].

It is worth noting that many receptor structures and ensembles are capable of operating in both modes. In particular, FA (focal adhesions)-proteins, membrane G-protein coupled receptors (GPCRs), dense intercellular AJs contacts (adherens junctions), and most of the stress-activated channels (SACs) [21,74,75,76,128,131,132,133,134,135,137,138,139,147,148,149,150,151,152,153,154,155,156,157,158]. Therefore, the division into inside-out and outside-in is more valid for how a particular response of the same structure is regulated than for a specific structure.

SACs include numerous ion channels of varying degrees of specificity belonging to the K2P potassium channel (TREK-1, TREK-2), DEG-ENa-ASIC, TRP, and piezo channel families [142,143,159,160,161]. There are two models of regulation of ion channel opening under the action of mechanical load: “force-from-lipid” and “force-from-filament”. According to the first “force-from-lipid” model, the response to mechanical stimulation follows the scheme: external force → reorganization of the lipid environment of receptors → conformational changes of the receptor → opening/closing of the channel or another way of signal transduction [149,154,157]. According to the second model, ion channels, such as part of SACs, are physically bound to proteins of the cytoskeleton and/or extracellular matrix. In this case, the channel opening scheme would be as follows: external force → tension changes of the extracellular or intracellular matrix → conformational change of the channel. It is assumed that changes in the tension of the cell matrix cause reorganization of membrane lipids. The latter further facilitates signal transmission from an open channel to a still closed channel [151,152]. Thus, signal transduction from an open mechanosensitive channel to long distances along the membrane can be achieved.

Further signal transduction (mechanotransmission and mechanoresponse steps) can occur through several signaling pathways. It is noteworthy that the type of receptors involved and the further signal transduction pathway are determined by the type of acting mechanical action [31]. The whole variety of mechanical impacts on the cellular level is finally reduced to compression, tension, shear, and their combinations.

Cell compression is perceived by receptors from the integrin families, piezo channels, and TRP- and FA-receptors that trigger Wnt/β- and YAP/TAZ-dependent signaling pathways [162,163,164,165]. Tension is perceived by a similar set of receptors (integrins, TRPs, FAs) with additional cytoskeletal involvement. However, the signaling pathways triggered are different and include Rho-ROCK, MAPK, and ERK kinase cascades [166,167]. Shift (shear stress) is recognized by a wider range of receptors than compression and tension. A shift-sensing system including GPCRs, tyrosine kinase receptors, lipid rafts, gap junctions, and cell contacts, triggering both Wnt/β-dependent and kinase signaling pathways (Rho-ROCK, MAPK ERK) [168,169,170,171,172,173,174,175,176].

Mechanotransduction during US treatment should be separately mentioned. The main acting factor in this case is the shock wave formed by the cavitation of microbubbles [168]. Acoustic stimulation activates TRPs, piezo channels, and cell contact areas. Signaling pathways include cascades involving Rho-ROCK, MAPK, ERK, Wnt, ESCRT, Epac-Rap, YAP, and PI3K/Akt [177]. The reader can learn more about each of these signaling pathways in the cited reviews. Another mechanism involves action on cell membrane lipids [178].

### 3.2. ROS Generation in Water Solutions

Water is subjected to mechanical action, not only by human will, but also by nature. For example, when water droplets fall on a solid surface during a rainstorm or when water falls from a waterfall. In this case, physicochemical parameters such as concentration of dissolved gases, hydrogen index, temperature, electrical conductivity [179], redox potential [89], and light scattering spectra in the near-ultraviolet region [83] change in water. Changes in some physicochemical properties of water after mechanical impact can persist for up to several days [180]. It has been found that mechanical impact leads to the formation of reactive oxygen species (ROS) [27]. ROS are extremely reactive compounds that, on the one hand, can damage biological macromolecules [181] and, on the other hand, play an important signaling and regulatory role in biological systems [182]. Of all ROSs, only hydrogen peroxide is a stable molecule. The other ROS species have short lifetimes [183]. This allows hydrogen peroxide to accumulate in the medium under prolonged exposure, which facilitates detection and allows us to work with large concentrations. In this connection, most of the works most often investigate the effect of a particular low-intensity physical factor on hydrogen peroxide generation. The generation of hydrogen peroxide under mechanical action seems to occur according to two main scenarios:

1. Activation of oxygen molecules dissolved in water;
2. Dissociation of water molecules and hydroxyl anions.

**Figure 1:**
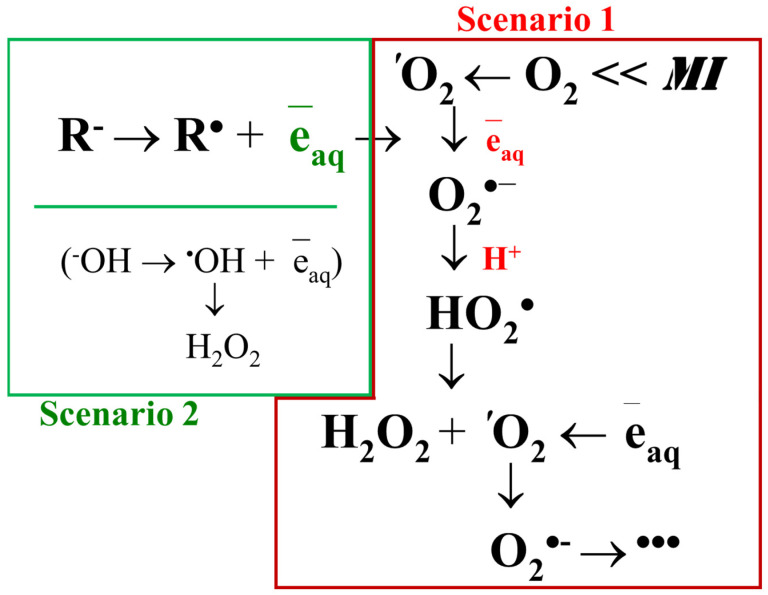
Scheme of reactions leading to the formation of hydrogen peroxide at mechanical impact on water, aqueous solutions, and aqueous colloids. MI is mechanical impact. Scenario 1: activation of molecular oxygen. Scenario 2: oxidation of anions.

The first scenario is related to the activation of molecular oxygen (Figure 1). It has been shown that under mechanical action, a part of the molecules of dissolved oxygen in water change from a triplet to a singlet state [184]. It is assumed that the transition of oxygen molecules from the triplet state to the singlet state occurs due to the impacts of dipoles [185]; it is also assumed that a local magnetic field can be generated under mechanical action, affecting the triplet-singlet transition [186]. More detailed views on the processes occurring with oxygen molecules dissolved in water under mechanical action can be found in studies [86,187,188]. Experimental validation of scenario 1 and its detailed discussions in studies [80,179]. The mechanical-induced generation of singlet oxygen in water was proofed by inhibitory analysis with sodium azide, NaN_3_ (singlet oxygen quencher). NaN_3_ inhibits mechanically induced ROS production (measured by luminol-dependent chemiluminescence) by 30%. These data indicate that singlet oxygen is involved in the generation of hydrogen peroxide during mechanical stress (30 Hz, 5 mm amplitude, 5 min) [80].

Singlet oxygen is further reduced to superoxide anion radical, which is protonated to hydroperoxide radical. The dismutation of hydroperoxide radicals produces hydrogen peroxide and molecular oxygen, in some cases in the singlet state; the cycle is closed. A similar scenario is observed in the generation of hydrogen peroxide upon exposure to visible radiation [189], infrared radiation [190], and heat [191]. The chemical transformations of ROS characteristic of this scenario are described in detail [192].

The second scenario is related to the oxidation of hydroxyl anions and/or the dissociation of water molecules. The possibility of the existence of such a process was theoretically predicted almost half a century ago [193]. This prediction has been criticized in terms of the energetics of the process [194]. It is believed that a quantum with an energy of about 5 eV is required to break the O-H bond in a water molecule [195]. The formation of hydroxyl radicals from water molecules has been observed under the action of ionizing radiation [196], plasma [197], and other high-energy effects, for example, during the optical breakdown of water [198]. In general, the scientific community was rather skeptical about the information concerning the generation of hydroxyl radicals from hydroxyl anion or water molecules under ‘weak’ non-ionizing effects, including mechanical ones, until the publication of theoretical works by Prof. Nowakowka’s group [199] and others. Five years ago, the phenomenon of hydroxyl radical generation under mechanical action was brilliantly experimentally confirmed by the example of the generation of micron-sized water droplets [90]. Today, it is assumed that the oxidation of hydroxyl anions is based on the mechanism of asymmetric charge of water droplets. When water is atomized, charged microdroplets are formed carrying excessive amounts of their own OH^−^ or H^+^ ions, which repel each other towards the droplet surface. The formation of ROS is attributed to limited hydration at the water-air phase interface [200]. It is assumed that both of the described scenarios occur in parallel during mechanical action in water. The efficiency of ROS generation in the first or second scenario mainly depends on such parameters as the frequency and amplitude of impact, conditions for the generation of micron-sized droplets during impact, and the concentration of molecular oxygen dissolved in water.

## 4. Dependence of the Magnitude of the Effects of Mechanical Impacts on Their Characteristics

### 4.1. Mechanical Influence

#### 4.1.1. Principal Conditions of Realisation of Effects

In the first stage, we evaluated the principal limits of the characteristics of the described mechanical effects. m/s^2^), and Characteristics we chose: frequency of vibration (Hz), acceleration that the sample acquires (sum duration of impact (s). Impact times, if expressed in hours or minutes, were converted to seconds. If periodic incubation was described (e.g., 5 times 100 s), the sum of the times was calculated and expressed in seconds (e.g., 500 s). Acceleration values were taken as initial values given in publications, or acceleration was calculated as the second derivative of the coordinates using the formula:

(1) x(t)=Asin(2πft),

(2) $$x^{\prime \prime }(t)=-4A\pi^{2}f^{2}\sin(2\pi ft)$$

(3) $${x^{\prime \prime }}_{\max}=4A\pi^{2}f^{2}$$

where *x*(*t*) is the sample coordinate, f is the frequency, *A* is the oscillation amplitude, and *x’’*_max_ is the maximum acceleration.

**Figure 2:**
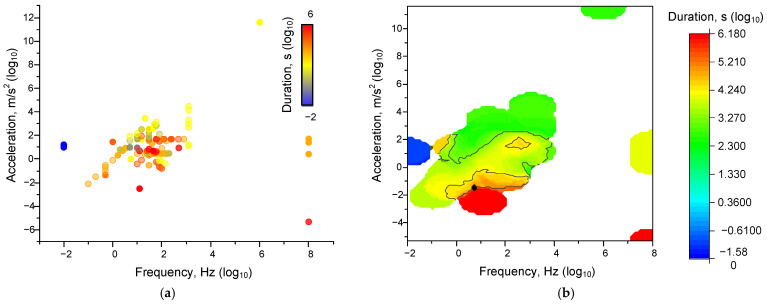
Dependences of the thresholds of the mechanical influence characteristics on all analysed systems: Dot plots (**a**) and 3D-map (**b**) of the characteristics ‘frequency-acceleration-duration’ at which effects were observed (irrespective of their magnitude). Each point corresponds to one set of published data (one row in Appendix A Table A1). A set of 339 published data sets was analyzed.

Frequency in cases of expression in rpm, etc., was converted into Hz. In this case, to determine the specified limits, we constructed three-dimensional distributions. It can be seen that the boundaries of applied frequencies, accelerations, and durations are quite strictly localized (Figure 2). Most effects were registered at frequencies from 0.01 to 500 Hz and at accelerations from 0.01 to 1000 m/s^2^. The duration of exposure required to achieve the effects increased as both frequency and acceleration decreased. Consequently, the effects of mechanical influence are realized when a certain “threshold” of transferred kinetic energy is overcome. This threshold is determined simultaneously by all three components: frequency, acceleration, and duration. At the same time, the values are partially interchangeable: low values of frequency and acceleration can be compensated by high duration and vice versa. Please note that Figure 2 shows not the magnitudes of the effects, but the threshold values of frequency, amplitude, and duration at which any effects were observed (taken as an “all-or-nothing” response). The quantitative values of the effects are already presented in the following figures.

#### 4.1.2. Dependences of Mechanical Effects on the Combination of Factors

The articles analyzed assessed a wide variety of effects of mechanical exposure: from the concentration of ROS and inorganic ions to the fraction of differentiated cells and apparent human muscle strength [26,51,79,86,104]. All values are expected to have different units of measurement. Thus, it is not possible to directly compare the data obtained in different articles. For this reason, we have converted all data into dimensionless quantities: effects were expressed as a percentage of the control values. The effect of mechanical influence can be expressed both as an increase in the value of any arbitrarily chosen parameter (e.g., an increase in cell proliferation) and a decrease (a slowdown in cell proliferation). Since we are not interested in the direction but numerically in the magnitude of the treatment facilitation effect, we decided to take all calculated percentages modulo the magnitude. We will hereafter refer to the resulting values as “relative effect moduli” or simply “effects”.

To estimate quantitative dependencies, we applied the approach we used and described earlier [201]. To assess the influence of the combination of conditions, we constructed dot plots in 3 axes. Further, we will denote the axes under consideration in the following order: “ordinate axis—abscissa axis—z (colirbar) axis”.

**Figure 3:**
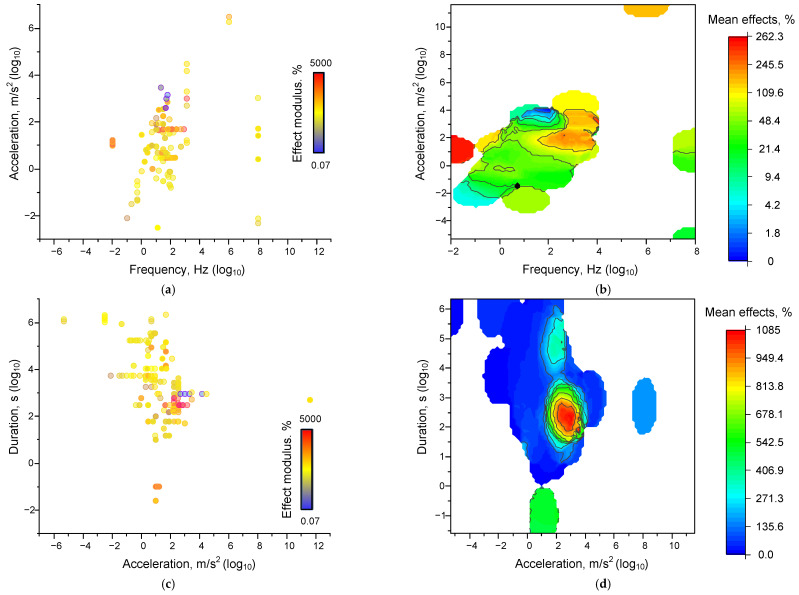

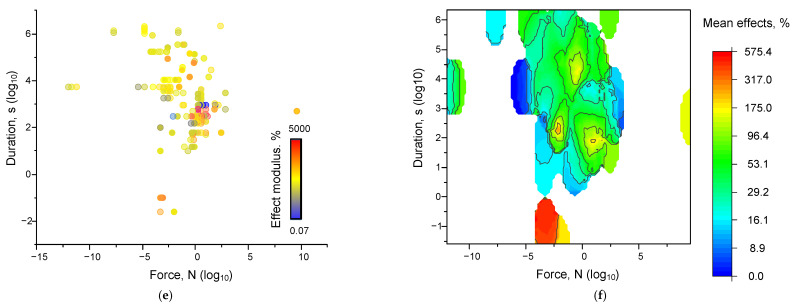
Dependences of the magnitude of the effects of mechanical influence on all analyzed systems: Dot plots of the characteristics ‘frequency-acceleration-effect’ (**a**), ‘acceleration-duration-effect’ (**c**), and ‘force-duration-effect’ (**e**). Each point corresponds to an individual parameter value in published papers. The color of the dot corresponds to the magnitude of the modulus of the effect relative to the control taken modulo. 3D maps of the distribution of the magnitude of the averaged effect sizes for the characteristics ‘frequency-acceleration-effect’ (**b**), ‘acceleration-duration-effect’ (**d**), and ‘force-duration-effect’ (**f**). Each point corresponds to one set of published data (row in Appendix A Table A1). A set of 339 published data sets was analyzed.

We evaluated the dependence of the magnitude of the effects of mechanical influence. The magnitude of the moduli of the relative effects of mechanical influence was weakly dependent on the frequency, acceleration, and duration of the influence. The most obvious dependence was found for the “acceleration-duration-effect” dependence (Figure 3c,d). We can see a clear enough boundary of acceleration and duration values, below which the effects of mechanical influence cease to be realized, which confirms the observed “threshold” effect described above. For the set of “frequency-acceleration-effect” characteristics, the dependence takes an unobvious form (Figure 3a,b). The separation into regions of 0.001–1 Hz and above 1 Hz is visible. In the first region, the effects are registered relatively rarely. In the second region, most of the effects are registered. There is a general tendency for the magnitudes of mechanical effects to increase with increasing acceleration and/or frequency. However, the magnitudes of the effects depend on the values of acceleration and frequency, not monotonically. The region between frequencies of 10–100 Hz and accelerations of 10–50 m/s^2^, at which the magnitudes of the effect moduli are the largest, can be seen. In the investigated works, there are often additional factors that are difficult to take into account (sample mass, laboratory temperature, and other microclimate features). In order to minimize the contribution of extraneous factors, we have attempted to calculate the force of the mechanical effect using the standard formula:

(4) F=ma,

where *F* is the force acting on the sample, *m* and *a* are the mass and the acceleration of the sample, respectively (taken from published papers or calculated from published data).

In the case of studies where the mass of the sample was clearly described, the values given by the authors were used. If the volumes of aqueous solutions studied were described, the assumption was made that *m* ≈ *V*, since the density of water under standard conditions is 0.99707 g/cm^3^. If “whole body mass” was specified in the in vivo experiments, the average mass of the animals or volunteer subjects specified in the paper was taken as mass. If the study was performed on an isolated limb or tail of the animal, the literature data on the mass of the indicated body parts were used [202,203].

We found that the shape of the dependence of the magnitude of the effect of mechanical impact on the combination of force and duration (Figure 3d,e) is similar to the shape of the acceleration-duration-effect relationship, but the point cloud is more widely distributed.

The increase in the “spread” of the dots indicates the dependence of the magnitude of the mechanical action on the mass of the sample, which determines the acceleration threshold required to achieve a certain value of acceleration. It is noteworthy that we found inhomogeneously distributed maxima of the effect moduli values within the force ranges from 0.0001 to 1000 N and from 1 s to several days. The presence of well-defined regions of frequency and acceleration values at which the effects are observed is consistent with the data of mathematical modeling and the theory of potential energy surfaces [204]. The form of the double force-duration or acceleration-duration dependence is similar to the previously described dependence obtained during the action of shear stress on non-neurons and myoblasts [153,155].

**Figure 4:**
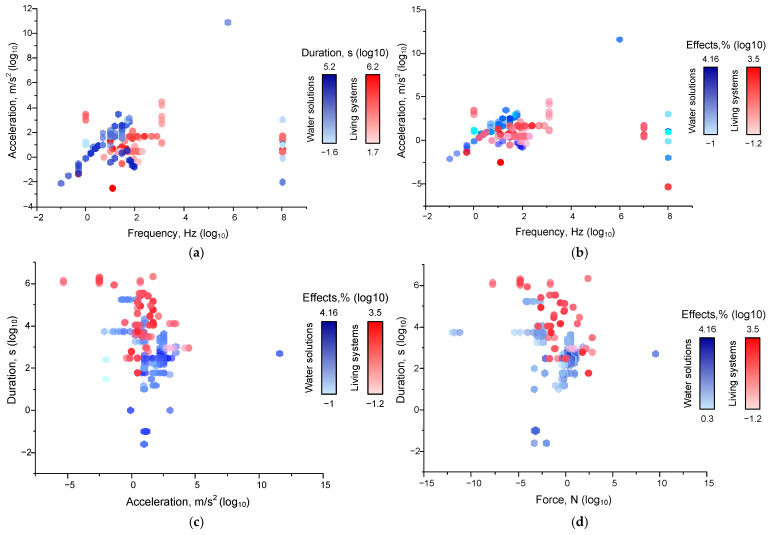
Dependences of the effects of mechanical impact on living (red circles) and non-living (blue hexagons) systems: (**a**) thresholds of the values of frequency, acceleration, and duration, at which the effects of mechanical impact were observed, and dependence of ‘frequency-acceleration-effect’ (**b**), ‘acceleration-duration-effect’ (**c**) and ‘force-duration-effect’ (**d**) (see Table 1) on living (red circles) and non-living (blue hexagons) systems. The legend shows color scales with the maximum and minimum values marked for each group compared. Each point corresponds to one set of published data (one row in Appendix A Table A1). A set of 339 published data sets was analyzed.

At the next stage, we attempted to answer the question: are there any fundamental differences between the magnitudes of the effects of mechanical influence from its characteristics, estimated earlier, for living and nonliving systems? For this purpose, we divided the data we analyzed into two groups. For the division, we used the classical paradigm of biology that the cell is the basic structural and functional unit of all life forms, and the manifestation of all the properties of living things begins at the level of the whole cell [205]. In the first group, we referred to the literature data on non-living systems: purified water, buffer solutions, isolated proteins in aqueous solutions, solutions of monomers in water, etc. The results on living systems included data obtained on cell lines, animals, and humans (Figure 4).

The threshold limits for non-living systems are broader than for living systems (Figure 4a) and are shifted to lower frequencies and accelerations. This is partly due to methodological limitations that are imposed on biological studies. However, this may explain the use of higher frequencies, accelerations, and durations. Reducing the above parameters should not cause methodological difficulties and/or confirmation of living subjects. In addition, mechanical action needs a much longer exposition to achieve an effect when acting on living systems than non-living systems. The difference in action time is on average 10–15 times. This phenomenon can be explained by fundamental differences in the way systems respond. “Response” of non-living systems to mechanical impact is provided exclusively by physical and chemical processes (energy dissipation, free radical reactions, etc.). Whereas, the responses of living systems are often mediated by specific receptors that trigger intracellular signaling cascades of events with some delay (see above). Receptor-mediated responses can take from fractions of seconds to minutes, hours, or even longer. Responses of living systems are also characterized by significant time delays due to the speed of the processes regulated by the cell or organism. Protein synthesis requires time delays of more than an hour, cell division—days, and tissue regeneration and remodeling—days or weeks.

Such a difference in “sensitivity” can be explained by the presence of homeostasis in living systems: up to a certain threshold, living systems can compensate for the effects of mechanical influence. According to our evaluation, the limits of metabolic compensation of mechanical impact lie in the areas of frequencies up to 10 Hz and accelerations up to 1 m/s^2^ with durations not exceeding 100 s.

In the next step, we assessed the dependence of effect sizes on the combination of force and duration (Figure 4b). We also found a significant difference in the limits of force and duration values at which the effects are registered. For non-living systems, a force of 10^−10^ N or a duration of 0.01 s is sufficient for the development of effects. Whereas, for living systems, forces of at least 0.001 N and a duration of ~100 s are required. This difference can be explained by the ability of living systems to actively self-regulate and maintain homeostasis. With increasing force and/or duration, the effects of mechanical impact on nonliving systems grow relatively monotonically. The largest effect sizes (up to 2–4 orders of magnitude) for aqueous solutions are observed in the range of forces >10 N and durations of 100–1000 s. For living systems, the dependence of the magnitude of effects on force and duration is not obvious. We can detect a region of increased effect values between 0.001 and 10 N and 300 and 10,000 s, where the largest number of effects with the largest moduli are observed. However, the presence of this region may be due more to the convenience of studying cells and animals in the laboratory under these conditions. Thus, the effects of mechanical impact on non-living systems depend on a combination of factors, the leading ones being acceleration, force, and duration. In the case of non-living systems, there is an increase in the magnitude of the effect from the post force and/or duration. For living systems, a threshold mechanism of mechanical action close to the “all or nothing” principle and/or a complex character of dependence of the efficiency of mechanical action on its characteristics is most likely.

#### 4.1.3. Correlations of Effects from Individual Parameters

**Figure 5:**
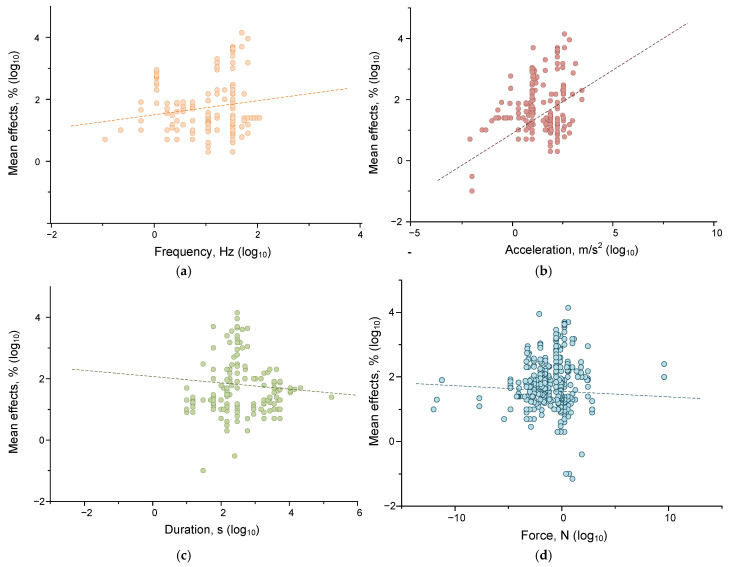
Dependences of the moduli of the effects of mechanical impact on water and aqueous solutions on the magnitude of frequency (orange) (**a**), acceleration (brown) (**b**), duration (green) (**c**), and force (cyan) (**d**). Each point corresponds to one set of published data (one row in Appendix A Table A1). The dash lines indicate fitting curves. A set of 192 published data sets was analyzed.

**Figure 6:**
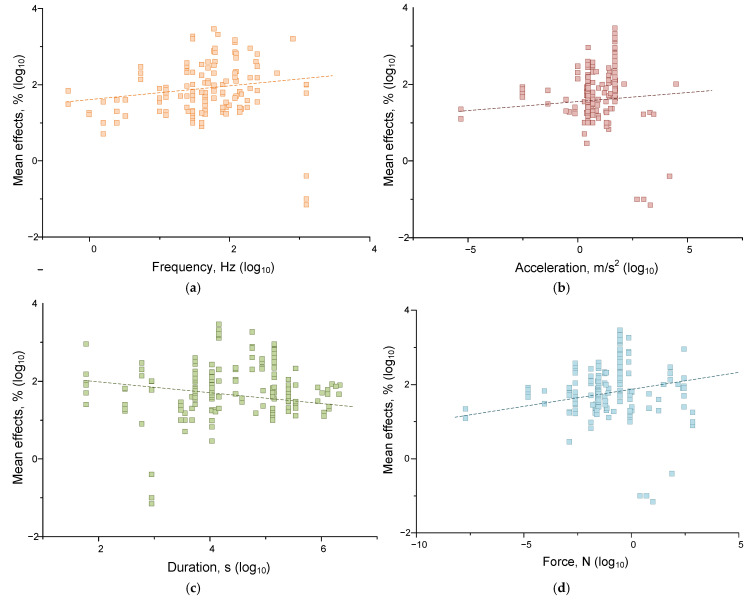
Dependences of the moduli of the effects of mechanical impact on living systems (cells and organisms) on the magnitude of frequency (frequency) (**a**), acceleration (brown) (**b**), duration (green) (**c**), and force (cyan) (**d**). The dash lines indicate fitting curves. Each point corresponds to one set of published data (one row in Appendix A Table A1). A set of 174 published data sets was analyzed.

At the next stage of the analysis, we tried to answer the question: which of the parameters of mechanical impact determines the magnitude of their effects to a greater extent? For this purpose, we evaluated the correlations between the magnitude of the effect of mechanical impact and its frequency, acceleration, force, and duration. Previously, we found that living and non-living systems respond differently to mechanical forcing, so in evaluating the correlations, we divided all evaluated responses into those of non-living (Figure 5) and living (Figure 6) systems. The strength, direction, and statistical significance of correlations were assessed using the Spearman criterion (Table 3).

We found that the magnitude of the effects of mechanical influence in the case of living systems depended on frequency, acceleration, and force. Significant correlations of the effect size with frequency (correlation strength 0.173 at *p* < 0.05), acceleration (correlation strength 0.253 at *p* < 0.001), and force (correlation 0.195, *p* < 0.05) were found. For non-living systems, the situation was somewhat different. Significant correlations were found between the magnitude of the effect size of mechanical impact and the frequency (*p* < 0.01), acceleration (*p* < 0.01), and duration (*p* < 0.001) of mechanical impact. However, the correlations found were of low strength: ~0.18 for the frequency-effect pair, ~0.22 for the acceleration-effect, and ~−0.26 for the duration-effect. Thus, mechanical effects with higher values of frequency and acceleration and the shortest durations should be most effective for water and aqueous solutions. It is noteworthy that living systems are more sensitive to acceleration and force than non-living systems. This can be explained by the ability of living systems to actively receive force (compression, stretching, shear stress) due to specialized receptor structures [128,129,130]. At the same time, nonliving systems are more sensitive to the duration of impact, which may indicate the contribution of mechanisms for maintaining homeostasis in living systems.

### 4.2. Dependence of the Magnitude of Ultrasound Effects on Its Characteristics

**Figure 7:**
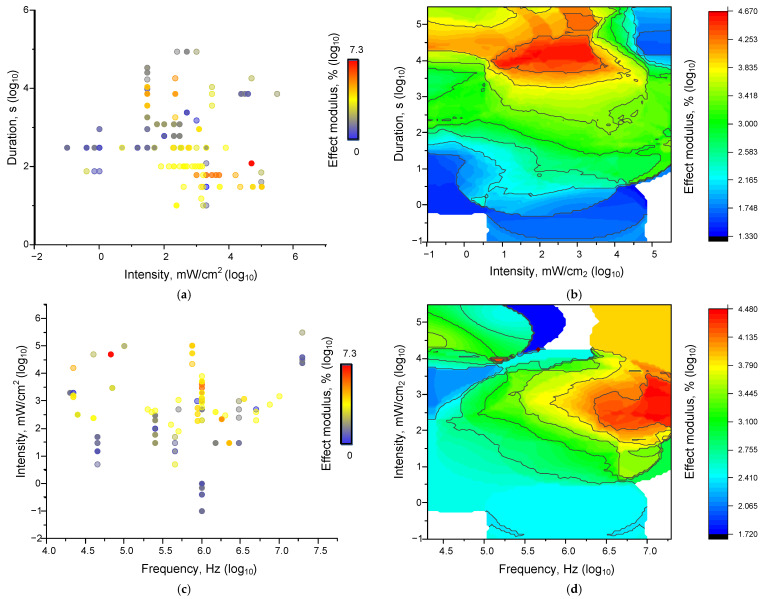
Dependences of the magnitude of ultrasonic influence effects on all analyzed systems: Dot plots of the characteristics “intensity-duration-effect” (**a**), “frequency-intensity-effect” (**c**). Each dot plot corresponds to an individual parameter value in published papers. The color of the point corresponds to the magnitude of the modulus of the effect relative to the control taken modulo. 3D maps of the distribution of the magnitude of the averaged effect sizes for the characteristics “intensity-duration-effect” (**b**), “frequency-intensity-effect” (**d**). In each variant, 225 results described in the literature were analysed.

To assess the dependence of ultrasound (US) effects on its characteristics, we applied an approach similar to that described in Section 4.1.2 and Section 4.1.3. We chose the frequency, intensity, and duration of exposure as the main characteristics of US treatment (Figure 7).

The characteristics of ultrasound exposure used in most of the analysed works lie in the frequency ranges from 10^4^ to 10^7^ Hz, and intensity from 0.1 mW/cm^2^ to 100 W/cm^2^. The durations are from 10 s to several hours. The bulk of the effects with high values are concentrated in the frequency range from 0.1 to 1.0 MHz (Figure 7). Increasing both frequency and intensity enhances the effects of ultrasound (US) on living and non-living systems (Figure 7). The contribution of frequency appears to be more significant than that of power. Similarly, the contribution of duration appears to be more significant than that of intensity. A threshold duration of 1000 s is observed, after which the magnitude of the effects increases significantly, regardless of power.

#### Correlations of Ultrasound Effects on Individual Parameters

**Figure 8:**
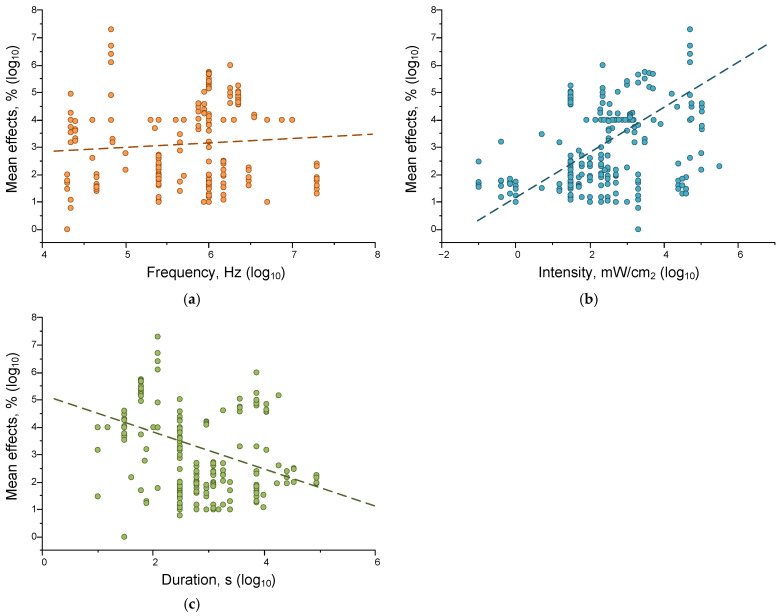
Correlations of frequency (orange) (**a**), intensity (cyan) (**b**), and duration (green) (**c**) of ultrasonic exposure and magnitude of effects on all analyzed systems. In each variant, 225 results published in the literature were analyzed. The dash lines indicate fitting curves.

When assessing the correlations between the magnitudes of the effects of the US on living and non-living systems, we found no differences, so we calculated Spearman correlation coefficients for the whole sample of analyzed data (Figure 8 and Table 4).

We found statistically significant correlations between the strength of the effect of ultrasound exposure and the intensity (correlation strength 0.38, *p* = 3.60925 × 10^−9^) and duration of exposure (correlation strength −0.27719, *p* = 2.8004 × 10^−5^). The positive correlation between the magnitude of US effects and its power seems more logical and does not require further explanation. The absence of a significant correlation between the magnitude of the effects of ultrasound (US) and its frequency indicates that frequency plays a less pronounced role in regulating the magnitude of US effects. Additionally, the frequency range used is narrower (3.5 orders of magnitude) than the duration and intensity ranges (5 and 7 orders of magnitude, respectively). The observed negative relationship between US exposure duration and effects can be explained by the fact that shorter exposure times were investigated at higher intensities, and vice versa (Figure 7a).

## 5. Limitations and Prospects

As can be seen from the Appendix A, there are more than a dozen (vibrations, shocks, stirring in different modes, falling, etc.) fundamentally different protocols of mechanical influence on aqueous solutions and living systems. For each of the effects, there may be critical conditions for the realization of effects that are not important for the other effects. For example, frequency is not applicable to falling, and height above the floor is not important in vibration. The difference in methods causes a difference in the information provided by the authors (see Appendix A), so the task of combining and comparing data becomes more difficult.

We have attempted to “arrive at a common denominator” when evaluating heterogeneous mechanical effects in the form of acceleration or force calculations. This approach allowed us to discover several interesting regularities, but it is not without disadvantages. In particular, when calculating the acceleration, we made the assumption that the oscillations are sinusoidal. However, we cannot guarantee that in all the analyzed articles the oscillations were sinusoidal. When calculating the force, we also used approximate mass values, since the authors’ exact mass values may simply not have been measured.

However, the approach we used was sufficient to find a number of generalizable acceleration-duration-effect and force-duration-effect patterns, and we also found differences between the responses to mechanical actions of living and non-living systems.

Two approaches can be suggested as future directions for research on this topic. The first is to deepen the analysis of already available data with the involvement of new methods of data analysis, for example, the principal component method, and the calculation of multiple correlation coefficients.

When planning future experiments, we would recommend limiting the number of measured effects in favor of expanding the number of exposure options. It is obligatory to perform adequate sham controls at all stages of the experiment [206]. In addition, when planning experiments (including those in other fields, not only mechanobiology) and interpreting the obtained data, it is necessary to take into account the possible effects of vibration and other influences on experimental samples, especially in vivo experiments.

The processes of cellular mechanotransduction are fundamentally different from those of solution mechanochemistry. In particular, actin-dependent transduction of mechanical signals in the cell is 40 to 50 times faster than diffusion of chemical reaction products [207,208]. However, as follows from the cited articles, mechanical action can affect the conformational states of proteins and enzymatic action in aqueous solution [91,92,209]. We proceed from the position that protein mechanochemistry, albeit altered, also occurs in the cytosol as in aqueous solutions, independent of signaling systems. Consequently, in a broad sense, the response of living systems to mechanical action will be the sum of aqueous solution mechanochemistry and mechanotransduction at the cellular level. Therefore, these processes are discussed together in this review.

We consider the use of mechanical effects for stem cell differentiation, more detailed in vivo studies, catalysis of chemical reactions in solutions, and nanobubbles as promising directions for future research.

## 6. Conclusions

Despite the diversity of mechanical effects, their characteristics can be summarised into a few key ones: frequency, acceleration, force, and duration. The magnitude of the effects of mechanical actions on living and non-living systems depends differently on the type of system affected. For non-living systems, there is a significant dependence of the response on the magnitude of acceleration, frequency, and duration of the impact. Responses of living systems depend on the magnitudes of acceleration, frequency, and force, but not duration. The magnitude of the effect changes insignificantly with the growth of these parameters of mechanical influence. Ultrasonic action depends on intensity and duration, both in aqueous solutions and in living organisms. Aqueous solutions require lower values of frequency and acceleration during mechanical action than living systems to initiate a ‘response’. This is due to the ability of living systems to maintain homeostasis. Notably, living systems are more sensitive to acceleration and force than non-living systems. This can be explained by the living system’s ability to actively receive force (compression, stretching, and shear stress) due to specialized receptor structures. At the same time, non-living systems are more sensitive to the duration of impact. These differences may indicate the contribution of mechanisms of homeostasis maintenance in living systems.

## Figures and Tables

**Table 1:** Effect of mechanical impacts on different levels of living systems.

System	Level	Reaction	Benefit	References
Cardiovascular	Molecular	Change Ca^2+^ responses to AChR ligands	↓ or ↑ depend on conditions	[70,71]
	Cells	Micro-damage of endothelial cells damage	↓	[41]
		Calcium homeostasis of endotheliocytes changes	↑	[70,71]
	Tissues	Number of capillaries, venules, and arterioles reduction	↓	[20]
		Smooth muscle work of microvessels	↑	[40]
	Organisms	HR and blood pressure, Raynaud’s syndrome, etc.	↓	[11,39]
Immune and blood cells	Molecular	NFAT activity, IL-6 synthesis, ROS production, and t-CPK activity increasing	↓	[40,41,42,43]
		Decreasing the synthesis of pro-inflammatory IFN-β, RANKL, and TNF-α, increased oxygen consumption, and gene expression of glycolysis enzymes and transporters (Hk2, GLUT1)	↑	[62,66,72]
	Cells	Erythrocytes hemolysis rate increasing	↓	[57]
		T-cell proliferation altered, ‘neutrophil respiratory burst’ (ROS productions), reduction of differentiated dendritic cell proliferation	↓ or ↑ depend on frequency and conditions	[32,33,56,72]
	Tissues	Immune memory and antitumor activity of dendric cells	↑	[72]
	Organisms	Anti-inflammatory effect	↑	[63]
		Proinflammatory effect	↓ or ↑ depend on conditions	[32,33]
Musculoskeletal	Molecular	TRAP-dependent pathway activation, increasing of ACAN and SOX9 expression, increasing of collagen content	↑	[44,45,59,67]
		P1NP and CTx concentration increase, osteogenesis regulators MMP 2 and OSX expression decreasing. Decreasing of osteocalcin levels, Runx2 and Osx gene expression, synthesis of osteoarthritis markers collagen II and aggrecan	↓	[26,28,46,47,49,50,58,64,65]
	Cells	Osteoclast proliferation, differentiations and activity increasing, increasing of differentiation of primary fibro chondrocytes	↑	[26,44,45,59,60,61]
		The viability of osteoblasts decreases; decrease of osteoblast differentiation	↓	[26,58]
	Tissues	Improving bone mechanical characteristics and bone structure	↑	[28,44,45]
		Bone tissue damage	↓	[42,43]
	Organisms	Osteoporosis markers P1NP and CTx levels	↓	[46,47]
		Improvement bone-implant contact, obesity decreasing, and muscle strength increasing	↑	[28,48,51]
Respiratory	Organisms	Inspiratory flow rate, inspired volume, and expired volume	↑	[52]
Nervous	Molecular	Increasing the expression of the IGF-I factor	↑	[54]
	Cells	Improve of myelinization	↑	[53]
Other	Molecular	Increasing in A cytochrome P450 monooxygenase CYP1B1 expression and metabolic activity changes	↑	[69]
	Cells	Stem cell proliferation inhibition	↓	[25]

↑ and ↓ indicate possible positive or negative effects on organisms, respectively.

**Table 2:** Effect of US impacts on different levels of living systems.

System	Level	Reaction	Benefit	References
Cardiovascular	Molecular	Enhancing VEGF secretion	↑	[102]
	Tissues	Enhance angiogenesis		[102,106]
Immune and blood cells	Molecular	Secretion of proinflammatory cytokines IL-1β, IL-8	↑	[102]
		Increasing the number of chromosomal aberrations	↓	[111]
	Cells	Induction of Ca^2+^ influx and platelet aggregation; induction of apoptosis of leucocyte cells	↓	[108,109,110,111,112]
	Organisms	inhibition of pro-inflammatory reactions	↑ or ↓ depend on situation	[113,115]
Musculoskeletal	Molecular	Increased expression of collagen, expression of myogenin, Pax7, COX2	↑	[102,113,115]
	Cells	Increasing of myocyte proliferation, myoblast differentiation	↑	[97,98,104]
	Tissues	Increasing of length of myotubes and the number of cells fused into one myotube, and regeneration of myofibrils	↑	[97,98,104,113,114]
Nervous	Molecular	Enhanced expression of markers Alk, Cenpf, Pcdh17 and actin	↑	[116]
	Cells	Promotions of proliferation and differentiation of neurons, formation of outgrowths	↑	[116]
Other	Molecular	Acceleration of drug delivery, EGFb regulatory factor expression increasing, change Cyclin D1/β, Cyclin B1/β, Cyclin E1/β, and Cyclin A1/β levels	↑	[99,102,103,105]
		Accelerate DNA plasmid transfer in bacterial cells	↑	[119]
		Plants photosynthesis inhibition	↓	[120]
	Cells	ER microstructure damage	↓	[100]
		Stem cell differentiation and proliferation increasing	↑	[102,103,104,107]
		Destruction of bacterial spores and cells	↓	[117,118]

↑ and ↓ indicate possible positive or negative effects on organisms, respectively.

**Table 3:** Spearman correlation coefficients calculated for mechanical effects.

Coefficients	Mechanical Impact Parameters	Effect log10	
		In Vivo	In Vitro
Spearman’s correlation	frequency, Hz (log_10_)	0.173	0.196
	acceleration, m/s^2^ (log_10_)	0.253	0.263
	duration, s (log_10_)	0.052	0.11407
	force, N (log_10_)	0.195	−0.06187
*p*-value	frequency, Hz (log_10_)	0.02344	0.01622
	acceleration, m/s^2^ (log_10_)	7.76 × 10^−4^	0.00113
	duration, s (log_10_)	0.49627	0.166
	force, N (log_10_)	0.01341	0.45196

The color scale in Spearman’s correlation corresponds to smaller negative (red) and larger positive (blue) values of Spearman’s Corr. The color scale in the *p*-value section shows significant (green, *p* < 0.05) and insignificant (orange, *p* ≥ 0.05) correlations.

**Table 4:** Spearman correlation coefficients between effect size and KM characteristics.

Coefficients	US Impact Parameters	Effect log10
Spearman’s correlation	frequency, Hz (log_10_)	0.1086
	intensity, mW/cm^2^ (log_10_)_	0.38302
	duration, s (log_10_)	−0.27719
*p*-value	frequency, Hz (log_10_)	0.10657
	intensity, mW/cm^2^ (log_10_)_	3.60925 × 10^−9^
	duration, s (log_10_)	2.8004 × 10^−5^

The color scale in Spearman’s correlation corresponds to negative (red) and larger positive (blue) values of Spearman’s Corr. The color scale in the *p*-value section shows significant (green, *p* < 0.05) and insignificant (orange, *p* ≥ 0.05) correlations.

## Data Availability

All collected and calculated data are provided in the table Appendix A.

## Abbreviations

The following abbreviations are used in this manuscript:

ACAN	Aggrecan
AJ	Adherens junctions
Akt	RAC-alpha serine/threonine-protein kinase
ALP	Alkaline phosphatase
ASICs	Acid-sensing ion channels
BMDCs	Bone Marrow-Derived Dendritic Cell
BrdU	5-bromo-2′-deoxyuridine
BSA	bovine serum albumin
CD	Cluster of differentiation
Cenpf	Centromere protein F
COL2A1	collagen II type gene
CPK	creatine phosphokinase
CYP1B1	cytochrome P450 1B1 gene
DKK1	Dickkopf-related protein 1
DLS	Dynamic light scattering
DMEM	Dulbecco’s Modified Eagle Medium
DN	double-network
E.G7-OVA	T lymphoblast cancer cell line
EGF	Epidermal Growth Factor
EMR	Electromagnetic radiation
ER	Endoplasmic reticulum
ERK	Extracellular signal-regulated kinase
fMLF	N-Formylmethionine-leucyl-phenylalanine
GPCRs	G-protein coupled receptors
hAD-MSC	Human amnion-derived mesenchymal stem cells
hESC	Human embryonic stem cells
HEWL	Hen egg white lysozyme
Hk2	Hexokinase II gene
hPDLSC	Human periodontal ligament stem cells
IDO	Indoleamine 2,3-dioxygenase
IFN	Interferon
IGF-I	insulin-like growth factor 1
IgG	Immunoglobulin G
IL	Interleukin
LINC	Linker of Nucleoskeleton and Cytoskeleton
MAPK	Mitogen-activated protein kinase
MMP2	Matrix metalloproteinase-2
MSCs	Mesenchymal stem cells
MT-1a	Metallothionein 1A
NFAT	Nuclear factor of activated T-cells
OCN	Osteocalcin
OPG	Osteoprotegerin
OSX	Osterix protein
PAAm	polyacrylamide
Pcdh17	Protocadherin 17
PEG	poly(ethylene glycol)
PGE-2	Prostaglandin E 2
PHEMA	poly(2-hydroxyethyl methacrylate)
PI3K	Phosphoinositide 3-kinases
PLA2	phospholipase A2
PNaAMPS	poly(2-acrylamido-2-methylpropanesulfonic acid sodium salt)
PVS	poly(vinyl alcohol)
RANKL	Receptor activator of nuclear factor kappa-B ligand
ROS	Reactive oxygen species
RPMI	Roswell Park Memorial Institute
Runx2	Runt-related transcription factor 2
SACs	stress-activated channels
Slc2a1	major glucose transporter GLUT1 gene
SOX	Superoxide dismutase
TGF	Transforming growth factor
TNF	Tumor necrosis factor
TREK	Two-Pore Domain K+ channels
TRP	Transient receptor potential channels
US	Ultrasound
VEGF	Vascular Endothelial Growth Factor

## Appendix A

**Table A1:** Mechanical impact on water solutions and living systems.

#	Solution	Object	Impact Type	Concetration (If Applied)	Frequency. Hz	Amplitude (Value)	Amplitude (Units)	Mass. g	Time. s	Mesured Characterisctic	Effect. %	Ref.
1	Culture medium DMEM	human fibroblasts	Ultrasound	-	45,000	15	mW/cm^2^	5	300	Cells proliferation	+30%	[102]
2	Culture medium DMEM	human fibroblasts	Ultrasound	-	45,000	50	mW/cm^2^	5	300	Cells proliferation	+45%	[102]
3	Culture medium DMEM	human fibroblasts	Ultrasound	-	1,000,000	0.7	mW/cm^2^	5	300	Cells proliferation	+45%	[102]
4	Culture medium DMEM	human fibroblasts	Ultrasound	-	1,000,000	1	mW/cm^2^	5	300	Cells proliferation	+55%	[102]
5	Culture medium DMEM	human fibroblasts	Ultrasound	-	45,000	15	mW/cm^2^	5	300	Collagen expression	+45%	[102]
6	Culture medium DMEM	human fibroblasts	Ultrasound	-	45,000	50	mW/cm^2^	5	300	Collagen expression	+38%	[102]
7	Culture medium DMEM	human osteoblasts	Ultrasound	-	45,000	5	mW/cm^2^	5	300	Cells proliferation	+32%	[102]
8	Culture medium DMEM	human fibroblasts	Ultrasound	-	1,000,000	0.1	mW/cm^2^	5	300	Collagen expression	+49%	[102]
9	Culture medium DMEM	human fibroblasts	Ultrasound	-	1,000,000	0.4	mW/cm^2^	5	300	Collagen expression	+58%	[102]
10	Culture medium DMEM	human fibroblasts	Ultrasound	-	1,000,000	0.7	mW/cm^2^	5	300	Collagen expression	+53%	[102]
11	Culture medium DMEM	human osteoblasts	Ultrasound	-	45,000	30	mW/cm^2^	5	300	Cells proliferation	+36%	[102]
12	Culture medium DMEM	human osteoblasts	Ultrasound	-	1,000,000	0.7	mW/cm^2^	5	300	Cells proliferation	+46%	[102]
13	Culture medium DMEM	human osteoblasts	Ultrasound	-	1,000,000	1	mW/cm^2^	5	300	Cells proliferation	+38%	[102]
14	Culture medium DMEM	human osteoblasts	Ultrasound		45,000	15	mW/cm^2^	5	300	Collagen expression	+45%	[102]
15	Culture medium DMEM	human osteoblasts	Ultrasound	-	45,000	50	mW/cm^2^	5	300	Collagen expression	+37%	[102]
16	Culture medium DMEM	human osteoblasts	Ultrasound	-	1,000,000	0.1	mW/cm^2^	5	300	Collagen expression	+53%	[102]
17	Culture medium DMEM	human osteoblasts	Ultrasound	-	1,000,000	0.4	mW/cm^2^	5	300	Collagen expression	+38%	[102]
18	Culture medium DMEM	human osteoblasts	Ultrasound	-	1,000,000	0.4	mW/cm^2^	5	75	Secretion of IL-1β	+16	[102]
19	Culture medium DMEM	human osteoblasts	Ultrasound	-	1,000,000	0.7	mW/cm^2^	5	75	Secretion of IL-1β	+20%	[102]
20	Culture medium DMEM	human osteoblasts	Ultrasound	-	1,000,000	1	mW/cm^2^	5	75	Secretion of IL-1β	+17%	[102]
21	Culture medium DMEM	human osteoblasts	Ultrasound	-	450,000	30	mW/cm^2^	5	300	Secretion of IL-8	+55%	[102]
22	Culture medium DMEM	human osteoblasts	Ultrasound	-	1,000,000	0.4	mW/cm^2^	5	300	Secretion of IL-8	+60%	[102]
23	Culture medium DMEM	human osteoblasts	Ultrasound	-	1,000,000	0.7	mW/cm^2^	5	300	Secretion of IL-8	+70%	[102]
24	Culture medium DMEM	human osteoblasts	Ultrasound	-	45,0000	5	mW/cm^2^	5	300	Secretion of EGFb	+3000%	[102]
25	Culture medium DMEM	human osteoblasts	Ultrasound	-	45,0000	15	mW/cm^2^	5	300	Secretion of EGFb	+1500%	[102]
26	Culture medium DMEM	human osteoblasts	Ultrasound	-	45,0000	50	mW/cm^2^	5	300	Secretion of EGFb	+750%	[102]
27	Culture medium DMEM	human osteoblasts	Ultrasound	-	1,000,000	0.1	mW/cm^2^	5	300	Secretion of EGFb	+300%	[102]
28	Culture medium DMEM	human osteoblasts	Ultrasound		450,000	15	mW/cm^2^	5	300	Secretion of VEGF	+25%	[102]
29	Culture medium DMEM	human osteoblasts	Ultrasound		1,000,000	0.1	mW/cm^2^	5	300	Secretion of VEGF	+40%	[102]
30	Culture medium RPMI 1640	human monocytes	Ultrasound	-	45,000	15	mW/cm^2^	5	300	Secretion of IL-1β	+25%	[102]
31	Culture medium RPMI 1640	human monocytes	Ultrasound	-	45,000	30	mW/cm^2^	5	300	Secretion of IL-1β	+103%	[102]
32	Culture medium RPMI 1640	human monocytes	Ultrasound	-	45,000	50	mW/cm^2^	5	300	Secretion of IL-1β	+80%	[102]
33	Culture medium RPMI 1640	human monocytes	Ultrasound	-	45,000	15	mW/cm^2^	5	300	Secretion of VEGF	+25%	[102]
34	Culture medium RPMI 1640	human monocytes	Ultrasound	-	45,000	30	mW/cm^2^	5	300	Secretion of VEGF	+30%	[102]
35	Culture medium RPMI 1640	human monocytes	Ultrasound	-	45,000	50	mW/cm^2^	5	300	Secretion of VEGF	+35%	[102]
36	Culture medium RPMI 1640	human monocytes	Ultrasound	-	1,000,000	0.1	mW/cm^2^	5	300	Secretion of VEGF	+35%	[102]
37	Culture medium RPMI 1640	human monocytes	Ultrasound	-	1,000,000	0.4	mW/cm^2^	5	300	Secretion of VEGF	+15%	[102]
38	Culture medium RPMI 1640	human monocytes	Ultrasound	-	1,000,000	1	mW/cm^2^	5	300	Secretion of VEGF	+10%	[102]
39	Culture medium DMEM	Human amnion-derived mesenchymal stem cells (hAD-MSC)	Ultrasound	-	250,000	100	mW/cm^2^	2	600	Cells differentiation	+10%	[103]
40	Culture medium DMEM	Human amnion-derived mesenchymal stem cells (hAD-MSC)	Ultrasound	-	250,000	200	mW/cm^2^	2	600	Cells differentiation	+15%	[103]
41	Culture medium DMEM	Human amnion-derived mesenchymal stem cells (hAD-MSC)	Ultrasound	-	250,000	300	mW/cm^2^	2	600	Cells differentiation	+15%	[103]
42	Culture medium DMEM	Human amnion-derived mesenchymal stem cells (hAD-MSC)	Ultrasound	-	250,000	60	mW/cm^2^	2	1200	Cells differentiation	+15%	[103]
43	Culture medium DMEM	Human amnion-derived mesenchymal stem cells (hAD-MSC)	Ultrasound	-	250,000	100	mW/cm^2^	2	1200	Cells differentiation	+15%	[103]
44	Culture medium DMEM	Human amnion-derived mesenchymal stem cells (hAD-MSC)	Ultrasound	-	250,000	200	mW/cm^2^	2	1200	Cells differentiation	+10%	[103]
45	Culture medium DMEM	Human amnion-derived mesenchymal stem cells (hAD-MSC)	Ultrasound	-	250,000	300	mW/cm^2^	2	1200	Cells differentiation	+11%	[103]
46	Culture medium DMEM	Human amnion-derived mesenchymal stem cells (hAD-MSC)	Ultrasound	-	250,000	30	mW/cm^2^	2	1800	Cells differentiation	+15%	[103]
47	Culture medium DMEM	Human amnion-derived mesenchymal stem cells (hAD-MSC)	Ultrasound	-	250,000	100	mW/cm^2^	2	600	Concentration of Cyclin D1/β	+60%	[103]
48	Culture medium DMEM	Human amnion-derived mesenchymal stem cells (hAD-MSC)	Ultrasound	-	250,000	200	mW/cm^2^	2	600	Concentration of Cyclin D1/β	+40%	[103]
49	Culture medium DMEM	Human amnion-derived mesenchymal stem cells (hAD-MSC)	Ultrasound	-	250,000	300	mW/cm^2^	2	600	Concentration of Cyclin D1/β	+45%	[103]
50	Culture medium DMEM	Human amnion-derived mesenchymal stem cells (hAD-MSC)	Ultrasound	-	250,000	60	mW/cm^2^	2	1200	Concentration of Cyclin D1/β	+100%	[103]
51	Culture medium DMEM	Human amnion-derived mesenchymal stem cells (hAD-MSC)	Ultrasound	-	250,000	100	mW/cm^2^	2	1200	Concentration of Cyclin D1/β	+90%	[103]
52	Culture medium DMEM	Human amnion-derived mesenchymal stem cells (hAD-MSC)	Ultrasound	-	250,000	200	mW/cm^2^	2	1200	Concentration of Cyclin D1/β	+80%	[103]
53	Culture medium DMEM	Human amnion-derived mesenchymal stem cells (hAD-MSC)	Ultrasound	-	250,000	300	mW/cm^2^	2	1200	Concentration of Cyclin D1/β	+70%	[103]
54	Culture medium DMEM	Human amnion-derived mesenchymal stem cells (hAD-MSC)	Ultrasound	-	250,000	30	mW/cm^2^	2	1800	Concentration of Cyclin D1/β	+110%	[103]
55	Culture medium DMEM	Human amnion-derived mesenchymal stem cells (hAD-MSC)	Ultrasound	-	250,000	100	mW/cm^2^	2	600	Concentration of Cyclin В1/β	+200%	[103]
56	Culture medium DMEM	Human amnion-derived mesenchymal stem cells (hAD-MSC)	Ultrasound	-	250,000	200	mW/cm^2^	2	600	Concentration of Cyclin В1/β	+380%	[103]
57	Culture medium DMEM	Human amnion-derived mesenchymal stem cells (hAD-MSC)	Ultrasound	-	250,000	300	mW/cm^2^	2	600	Concentration of Cyclin В1/β	+500%	[103]
58	Culture medium DMEM	Human amnion-derived mesenchymal stem cells (hAD-MSC)	Ultrasound	-	250,000	60	mW/cm^2^	2	1200	Concentration of Cyclin В1/β	+500%	[103]
59	Culture medium DMEM	Human amnion-derived mesenchymal stem cells (hAD-MSC)	Ultrasound	-	250,000	100	mW/cm^2^	2	1200	Concentration of Cyclin В1/β	+400%	[103]
60	Culture medium DMEM	Human amnion-derived mesenchymal stem cells (hAD-MSC)	Ultrasound	-	250,000	200	mW/cm^2^	2	1200	Concentration of Cyclin В1/β	+530%	[103]
61	Culture medium DMEM	Human amnion-derived mesenchymal stem cells (hAD-MSC)	Ultrasound	-	250,000	300	mW/cm^2^	2	1200	Concentration of Cyclin В1/β	+490%	[103]
62	Culture medium DMEM	Human amnion-derived mesenchymal stem cells (hAD-MSC)	Ultrasound	-	250,000	30	mW/cm^2^	2	1800	Concentration of Cyclin В1/β	+490%	[103]
63	Culture medium DMEM	Human amnion-derived mesenchymal stem cells (hAD-MSC)	Ultrasound	-	250,000	100	mW/cm^2^	2	600	Concentration of Cyclin Е1/β	+80%	[103]
64	Culture medium DMEM	Human amnion-derived mesenchymal stem cells (hAD-MSC)	Ultrasound	-	250,000	200	mW/cm^2^	2	600	Concentration of Cyclin Е1/β	+110%	[103]
65	Culture medium DMEM	Human amnion-derived mesenchymal stem cells (hAD-MSC)	Ultrasound	-	250,000	300	mW/cm^2^	2	600	Concentration of Cyclin Е1/β	+150%	[103]
66	Culture medium DMEM	Human amnion-derived mesenchymal stem cells (hAD-MSC)	Ultrasound	-	250,000	60	mW/cm^2^	2	1200	Concentration of Cyclin Е1/β	+200%	[103]
67	Culture medium DMEM	Human amnion-derived mesenchymal stem cells (hAD-MSC)	Ultrasound	-	250,000	100	mW/cm^2^	2	1200	Concentration of Cyclin Е1/β	+250%	[103]
68	Culture medium DMEM	Human amnion-derived mesenchymal stem cells (hAD-MSC)	Ultrasound	-	250,000	200	mW/cm^2^	2	1200	Concentration of Cyclin Е1/β	+200%	[103]
69	Culture medium DMEM	Human amnion-derived mesenchymal stem cells (hAD-MSC)	Ultrasound	-	250,000	300	mW/cm^2^	2	1200	Concentration of Cyclin Е1/β	+180%	[103]
70	Culture medium DMEM	Human amnion-derived mesenchymal stem cells (hAD-MSC)	Ultrasound	-	250,000	30	mW/cm^2^	2	1800	Concentration of Cyclin Е1/β	+190%	[103]
71	Culture medium DMEM	Human amnion-derived mesenchymal stem cells (hAD-MSC)	Ultrasound	-	250,000	100	mW/cm^2^	2	600	Concentration of Cyclin A1/β	+250%	[103]
72	Culture medium DMEM	Human amnion-derived mesenchymal stem cells (hAD-MSC)	Ultrasound	-	250,000	200	mW/cm^2^	2	600	Concentration of Cyclin A1/β	+100%	[103]
73	Culture medium DMEM	Human amnion-derived mesenchymal stem cells (hAD-MSC)	Ultrasound	-	250,000	300	mW/cm^2^	2	600	Concentration of Cyclin A1/β	+90%	[103]
74	Culture medium DMEM	Human amnion-derived mesenchymal stem cells (hAD-MSC)	Ultrasound	-	250,000	60	mW/cm^2^	2	1200	Concentration of Cyclin A1/β	+250%	[103]
75	Culture medium DMEM	Human amnion-derived mesenchymal stem cells (hAD-MSC)	Ultrasound	-	250,000	100	mW/cm^2^	2	1200	Concentration of Cyclin A1/β	+245%	[103]
76	Culture medium DMEM	Human amnion-derived mesenchymal stem cells (hAD-MSC)	Ultrasound	-	250,000	200	mW/cm^2^	2	1200	Concentration of Cyclin A1/β	+240%	[103]
77	Culture medium DMEM	Human amnion-derived mesenchymal stem cells (hAD-MSC)	Ultrasound	-	250,000	300	mW/cm^2^	2	1200	Concentration of Cyclin A1/β	+70%	[103]
78	Culture medium DMEM	Human amnion-derived mesenchymal stem cells (hAD-MSC)	Ultrasound	-	250,000	30	mW/cm^2^	2	1800	Concentration of Cyclin A1/β	+270%	[103]
79	Culture medium DMEM	Human amnion-derived mesenchymal stem cells (hAD-MSC)	Ultrasound	-	250,000	30	mW/cm^2^	2	1800	Cells proliferation	+180%	[103]
80	Culture medium DMEM	Mouse myoblasts (C2C12)	Ultrasound	-	500,000	500	mW/cm^2^	1	86,400	Cells proliferation	+90%	[104]
81	Culture medium DMEM	Mouse myoblasts (C2C12)	Ultrasound	-	1,000,000	500	mW/cm^2^	1	86,400	Cells proliferation	+95%	[104]
82	Culture medium DMEM	Mouse myoblasts (C2C12)	Ultrasound	-	3,000,000	500	mW/cm^2^	1	86,400	Cells proliferation	+150%	[104]
83	Culture medium DMEM	Mouse myoblasts (C2C12)	Ultrasound	-	3,000,000	1000	mW/cm^2^	1	86,400	Cells proliferation	+180%	[104]
84	Culture medium DMEM	Mouse myoblasts (C2C12)	Ultrasound	-	3,000,000	250	mW/cm^2^	1	86,400	Cells proliferation	+145%	[104]
85	Culture medium DMEM	Mouse myoblasts (C2C12)	Ultrasound	-	1,000,000	500	mW/cm^2^	1	2400	myotube width	+20%	[104]
86	Culture medium DMEM	Mouse myoblasts (C2C12)	Ultrasound	-	1,000,000	500	mW/cm^2^	1	2400	myotube length	+50%	[104]
87	Culture medium DMEM	Mouse myoblasts (C2C12)	Ultrasound	-	5,000,000	500	mW/cm^2^	1	2400	myotube length	+10%	[104]
88	Culture medium DMEM	Mouse myoblasts (C2C12)	Ultrasound	-	1,000,000	500	mW/cm^2^	1	2400	fusion index	+100%	[104]
89	Rat (trauma model)	in vivo	Ultrasound	-	870,000	1000	mW/cm^2^	250	1500	Total tissue protein content	−10%	[97]
90	Culture medium DMEM	Mouse myoblasts (C2C12)	Ultrasound	-	1,500,000	30	mW/cm^2^	10	7200	Cells proliferation	+18%	[113]
91	Culture medium DMEM	Mouse myoblasts (C2C12)	Ultrasound	-	1,500,000	30	mW/cm^2^	10	9600	Cells proliferation	+12%	[113]
92	Culture medium DMEM	Mouse myoblasts (C2C12)	Ultrasound	-	1,500,000	30	mW/cm^2^	10	7200	Expression of myogenin	+47%	[113]
93	Culture medium DMEM	Mouse myoblasts (C2C12)	Ultrasound	-	1,500,000	30	mW/cm^2^	10	9600	Expression of myogenin	+34%	[113]
94	Culture medium DMEM	Mouse myoblasts (C2C12)	Ultrasound	-	1,500,000	30	mW/cm^2^	10	16,800	Numbers of regenerating myofibers	+90%	[113]
95	Culture medium DMEM	Mouse myoblasts (C2C12)	Ultrasound	-	1,500,000	30	mW/cm^2^	10	25,200	Numbers of regenerating myofibers	+250%	[113]
96	Culture medium DMEM	Mouse myoblasts (C2C12)	Ultrasound	-	1,500,000	30	mW/cm^2^	10	33,600	Numbers of regenerating myofibers	+320%	[113]
97	Culture medium DMEM	Mouse myoblasts (C2C12)	Ultrasound	-	1,500,000	30	mW/cm^2^	10	25,200	Restoration of fast twitch strength	+150%	[113]
98	Culture medium DMEM	Mouse myoblasts (C2C12)	Ultrasound	-	1,500,000	30	mW/cm^2^	10	33,600	Restoration of fast twitch strength	+300%	[113]
99	Culture medium DMEM	Mouse myoblasts (C2C12)	Ultrasound	-	1,500,000	30	mW/cm^2^	10	25200	Restoration of tetanus strength strength	+90%	[113]
100	Culture medium DMEM	Mouse myoblasts (C2C12)	Ultrasound	-	1,500,000	30	mW/cm^2^	10	33600	Restoration of fast twitch strength	+100%	[113]
101	in vivo	Wistar rats	Ultrasound	-	1,000,000	1	mW/cm^2^	220	900	Muscle fibre diameter	+10%	[98]
102	in vivo	Wistar rats	Ultrasound	-	1,000,000	1	mW/cm^2^	220	900	BrdU positive cells count	+30%	[98]
103	Culture medium DMEM	Mouse myoblasts (C2C12)	Ultrasound	-	3,000,000	30	mW/cm^2^	5	900	Pax7 positive cells count	+60%	[115]
104	Culture medium DMEM	Mouse myoblasts (C2C12)	Ultrasound	-	3,000,000	30	mW/cm^2^	5	900	Expression of gene COX2	+80%	[115]
105	Culture medium DMEM	Mouse myoblasts (C2C12)	Ultrasound	-	3,000,000	30	mW/cm^2^	5	900	Proiflammatory cells count	−40%	[115]
106	Culture medium DMEM	BMDCs	Constant pressure from below	-	0	50,00050,000	Pa	5	950,400	Efficiency of glycolysis	−30%	[72]
107	Culture medium DMEM	BMDCs	Constant pressure from below	-	0	2000	Pa	5	950,400	Efficiency of glycolysis	−50%	[72]
108	Culture medium DMEM	BMDCs	Constant pressure from below	-	0	50,000	Pa	5	950,400	Lactase activity	−5%	[72]
109	Culture medium DMEM	BMDCs	Constant pressure from below	-	0	2000	Pa	5	950,400	Lactase activity	−60%	[72]
110	Culture medium DMEM	BMDCs	Constant pressure from below	-	0	50,000	Pa	5	950,400	overall rate curves of oxygen consumption rate	−70%	[72]
111	Culture medium DMEM	BMDCs	Constant pressure from below	-	0	2000	Pa	5	950,400	overall rate curves of oxygen consumption rate	−80%	[72]
112	Culture medium DMEM	BMDCs	Constant pressure from below	-	0	50,000	Pa	5	950,400	Expression of gene *Hk2*	−55%	[72]
113	Culture medium DMEM	BMDCs	Constant pressure from below	-	0	2000	Pa	5	950,400	Expression of gene *Slc2a1*	−96%	[72]
114	Culture medium DMEM	BMDCs	Constant pressure from below	-	0	2000	Pa	5	950,400	Cells proliferation	−95%	[72]
115	Culture medium DMEM	BMDCs	Constant pressure from below	-	0	50,000	Pa	5	950,400	Tumor size (11 days post E.G7-OVA injection) in WT mice injected with E.G7-OVA cells and WT BMDCs	−50%	[72]
116	Culture medium DMEM	BMDCs	Constant pressure from below	-	0	50,000	Pa	5	950,400	tumor weight (14 days post E.G7-OVA injection) in WT mice injected with E.G7-OVA cells and WT BMDCs	−85%	[72]
117	Culture medium DMEM	CD8+ T cells	Constant pressure from below	-	0	50,000	Pa	5	950,400	Immunology memory	+120%	[72]
118	Culture medium DMEM	CD4+ T cells	Constant pressure from below	-	0	50,000	Pa	5	950,400	Immunology memory	+110%	[72]
119	Water	Water	Shaking	-	30	12	mm	10	60	Chemiluminescence intensity	−20%	[21]
120	Water	Water	Shaking	-	30	2.3	mm	10	60	Chemiluminescence intensity	+25%	[21]
121	Water	Water	Shaking	-	10	12	mm	10	15	Decline rate of chemiluminescence intensity	−20%	[21]
122	Water	Water	Shaking	-	10	12	mm	10	60	Decline rate of chemiluminescence intensity	−25%	[21]
123	Water	Water	Shaking	-	30	2.3	mm	10	60	Decline rate of chemiluminescence intensity	+30%	[21]
124	Water	Water	Shaking	-	30	2.3	mm	10	60	Standard deviation of chemiluminescence intensity	+10%	[21]
125	Water	BSA	Shaking	1 mg/mL	30	2.3	mm	10	15	Chemiluminescence intensity	−20%	[21]
126	Water	BSA	Shaking	1 mg/mL	10	12	mm	10	15	Chemiluminescence intensity	−15%	[21]
127	Water	BSA	Shaking	1 mg/mL	10	12	mm	10	60	Chemiluminescence intensity	−10%	[21]
128	Water	BSA	Shaking	1 mg/mL	30	2.3	mm	10	15	Chemiluminescence intensity	−8%	[21]
129	Water	BSA	Shaking	1 mg/mL	30	2.3	mm	10	60	Chemiluminescence intensity	−20%	[21]
130	Water	BSA	Shaking	1 mg/mL	30	2.3	mm	10	15	Chemiluminescence intensity	−12%	[21]
131	Water	BSA	Shaking	1 mg/mL	30	2.3	mm	10	60	Chemiluminescence intensity	−20%	[21]
132	Water	BSA	Shaking	1 mg/mL	10	2.3	mm	10	15	Decline rate of chemiluminescence intensity	−18%	[21]
133	Water	BSA	Shaking	1 mg/mL	10	12	mm	10	15	Decline rate of chemiluminescence intensity	−17%	[21]
134	Water	BSA	Shaking	1 mg/mL	10	12	mm	10	60	Decline rate of chemiluminescence intensity	−16%	[21]
135	Water	BSA	Shaking	1 mg/mL	30	2.3	mm	10	15	Decline rate of chemiluminescence intensity	−25%	[21]
136	Water	BSA	Shaking	1 mg/mL	30	2.3	mm	10	60	Decline rate of chemiluminescence intensity	−16%	[21]
137	Water	BSA	Shaking	1 mg/mL	30	2.3	mm	10	60	Standard deviation of chemiluminescence intensity	−10%	[21]
138	Water	Human IgG	Alternating magnetic field	1 mg/mL	50	50	мкТл	10	300	Chemiluminescence intensity	+10%	[21]
139	Water	Human IgG	Alternating magnetic field	1 mg/mL	8	50	мкТл	10	300	Standard deviation of chemiluminescence intensity	+5%	[21]
140	Hanks’ balanced salts solution	Murine neutrophils (Male Balb/c mice weighing 22–25 g)	Alternating magnetic field	2.5 × 10^5^ cells/mL	12.6	0.01	мкТл		5400	fMLF-induced ROS generation	+45%	[32]
141	Hanks’ balanced salts solution	Murine neutrophils (Male Balb/c mice weighing 22–25 g)	Alternating magnetic field	2.5 × 10^5^ cells/mL	12.6	0.05	мкТл		5400	fMLF-induced ROS generation	+45%	[32]
142	Water	*Viscum album Quercus* L. 3. plant extract	Turbulent mixing	0.1% (10^−3^)	10	2	sm	10	150	local connected fractal dimension	−27%	[22]
143	Water	*Viscum album Quercus* L. 3. plant extract	Circular mixing	0.1% (10^−3^)	10	2	sm	10	150	local connected fractal dimension	+3%	[22]
144	Water	*Viscum album Quercus* L. 3. plant extract	Turbulent mixing	0.1% (10^−3^)	10	2	sm	10	150	mass fractal dimension	−9%	[22]
145	Water	*Viscum album Quercus* L. 3. plant extract	Circular mixing	0.1% (10^−3^)	10	2	sm	10	150	mass fractal dimension	+2%	[22]
146	Water	*Viscum album Quercus* L. 3. plant extract	Turbulent mixing	0.1% (10^−3^)	10	2	sm	10	150	lacunarity	+37%	[22]
147	Water	*Viscum album Quercus* L. 3. plant extract	Circular mixing	0.1% (10^−3^)	10	2	sm	10	150	lacunarity	+4%	[22]
148	Water	*Viscum album Quercus* L. 3. plant extract	Turbulent mixing	0.1% (10^−3^)	10	2	sm	10	150	ascending second moment	+30%	[22]
149	Water	*Viscum album Quercus* L. 3. plant extract	Circular mixing	0.1% (10^−3^)	10	2	sm	10	150	ascending second moment	−15%	[22]
150	Water	*Viscum album Quercus* L. 3. plant extract	Turbulent mixing	0.1% (10^−3^)	10	2	sm	10	150	contrast	−30%	[22]
151	Water	*Viscum album Quercus* L. 3. plant extract	Circular mixing	0.1% (10^−3^)	10	2	sm	10	150	contrast	−12%	[22]
152	Water	*Viscum album Quercus* L. 3. plant extract	Turbulent mixing	0.1% (10^−3^)	10	2	sm	10	150	Correlation	+30%	[22]
153	Water	*Viscum album Quercus* L. 3. plant extract	Circular mixing	0.1% (10^−3^)	10	2	sm	10	150	Correlation	+90%	[22]
154	Water	*Viscum album Quercus* L. 3. plant extract	Turbulent mixing	0.1% (10^−3^)	10	2	sm	10	150	Entropy	−3%	[22]
155	Water	*Viscum album Quercus* L. 3. plant extract	Circular mixing	0.1% (10^−3^)	10	2	sm	10	150	Entropy	+2%	[22]
156	Hanks’ balanced salts solution	Murine neutrophils (Male Balb/c mice weighing 22–25 g)	Alternating magnetic field	2.5 × 10^5^ cells/mL	0	0	0		3600	fMLF-induced ROS production primered by PMA	−21%	[33]
157	Hanks’ balanced salts solution	Murine neutrophils (Male Balb/c mice weighing 22–25 g)	Alternating magnetic field	2.5 × 10^5^ cells/mL	12.6	0.1	мкТл		3600	fMLF-induced ROS production primered by PMA	+67%	[33]
158	Hanks’ balanced salts solution	Murine neutrophils (Male Balb/c mice weighing 22–25 g)	Alternating magnetic field	2.5 × 10^5^ cells/mL	48.5	0.1	мкТл		3600	fMLF-induced ROS production primered by PMA	+635%	[33]
159	Hanks’ balanced salts solution	Murine neutrophils (Male Balb/c mice weighing 22–25 g)	Alternating magnetic field	2.5 × 10^5^ cells/mL	12.6	0.1	мкТл		2400	fMLF-induced ROS generation	+36%	[34]
160	Hanks’ balanced salts solution	Murine neutrophils (Male Balb/c mice weighing 22–25 g)	Alternating magnetic field	2.5 × 10^5^ cells/mL	48.5	0.1	мкТл		2400	fMLF-induced ROS generation	−19%	[34]
161	Water	IFNγ	Circular mixing	0.1 М	50	1	sm	10	50	Conductivity of the solution	+20%	[16]
162	Water	polyclonal antibodies to human IFNγ	Circular mixing	26 μg/mL	50	1	sm	10	50	Solurion EMR	+150%	[17]
163	Water	Lactose	Strokes	820 nM	21	300 (20)	g (CM)	24	1050	Luminescence at 550 nm	+100%	[24]
164	Water	Lactose	Strokes	820 нМ	21	300 (20)	g (CM)	24	525	Luminescence at 550 nm	+200%	[24]
165	20 mM histidine buffer.	antistreptavidin IgG1	Downfall	1 mg/mL	0	25.6	sm	1	0.025	Particle counts (protein aggregation?)	+150%	[91]
166	20 mM histidine buffer.	antistreptavidin IgG1	Downfall	1 mg/mL	0	51.2	sm	1	0.025	Particle counts	+300%	[91]
167	20 mM histidine buffer.	antistreptavidin IgG1	Downfall	1 mg/mL	0	76.8	sm	1	0.025	Particle counts	+1000%	[91]
168	20 mM histidine buffer.	antistreptavidin IgG1	Downfall	1 mg/mL	0	102.4	sm	1	0.025	Particle counts	+350%	[91]
169	20 mM histidine buffer.	antistreptavidin IgG1	Downfall	35 mg/mL	0	25.6	sm	1	0.025	Particle counts	+900%	[91]
170	20 mM histidine buffer.	antistreptavidin IgG1	Downfall	35 mg/mL	0	51.2	sm	1	0.025	Particle counts	+1100%	[91]
171	20 mM histidine buffer.	antistreptavidin IgG1	Downfall	35 mg/mL	0	76.8	sm	1	0.025	Particle counts	+1000%	[91]
172	20 mM histidine buffer.	human growth hormone	Downfall	35 mg/mL	0	102.4	sm	1	0.025	Particle counts	+800%	[91]
173	20 mM histidine buffer	human growth hormone	Downfall	1.75 mg/mL	0	102.4	sm	1	0.025	Particle counts	+60%	[91]
174	20 mM histidine buffer.	antistreptavidin IgG1	Downfall	1 mg/mL	0	25.6	sm	1	0.025	recovered from the walls of dropped vials following treatment with urea	+20%	[91]
175	20 mM histidine buffer	antistreptavidin IgG1	Downfall	1 mg/mL	0	76.8	sm	1	0.025	recovered from the walls of dropped vials following treatment with urea	+110%	[91]
176	20 mM histidine buffer.	antistreptavidin IgG1	Downfall	1 mg/mL	0	102.4	sm	1	0.025	recovered from the walls of dropped vials following treatment with urea	+180%	[91]
177	20 mM histidine buffer	antistreptavidin IgG1	Downfall	35 mg/mL	0	51.2	sm	1	0.025	recovered from the walls of dropped vials following treatment with urea	+60%	[91]
178	20 mM histidine buffer.	antistreptavidin IgG1	Downfall	35 mg/mL	0	76.8	sm	1	0.025	recovered from the walls of dropped vials following treatment with urea	+120%	[91]
179	20 mM histidine buffer	antistreptavidin IgG1	Downfall	35 mg/mL	0	102.4	sm	1	0.025	recovered from the walls of dropped vials following treatment with urea	+90%	[91]
180	20 mM histidine buffer	antistreptavidin IgG1	Downfall	1 mg/mL	0	25.6	sm	1	0.025	recovered from the walls of dropped vials following treatment with guanidine hydrochloride	+35%	[91]
181	20 mM histidine buffer.	antistreptavidin IgG1	Downfall	1 mg/mL	0	76.8	sm	1	0.025	recovered from the walls of dropped vials following treatment with guanidine hydrochloride	+30%	[91]
182	20 mM histidine buffer	antistreptavidin IgG1	Downfall	35 mg/mL	0	51.2	sm	1	0.025	recovered from the walls of dropped vials following treatment with guanidine hydrochloride	+200%	[91]
183	Water	*Echinacea*	succussion strokes	0.8 × 10^−2^ g/mL	1	20	sm	0.05	100	Characteristics of crystal shape after evaporation	+10%	[23]
184	Water	*Echinacea*	succussion strokes	0.8 × 10^−2^ g/mL	1	20	sm	0.05	10	Characteristics of crystal shape after evaporation	+8%	[23]
185	Water	*Baptisia*	succussion strokes	0.8 × 10^−3^ g/mL	1	20	sm	0.05	100	Characteristics of crystal shape after evaporation	−10%	[23]
186	Water	*Baptisia*	succussion strokes	0.8 × 10^−3^ g/mL	1	20	sm	0.05	10	Characteristics of crystal shape after evaporation	−10%	[23]
187	Water	*Baptisia*	succussion strokes	0.8 × 10^−4^ g/mL	1	20	sm	0.05	100	Characteristics of crystal shape after evaporation	+60%	[23]
188	Water	*Baptisia*	succussion strokes	0.8 × 10^−4^ g/mL	1	20	sm	0.05	10	Characteristics of crystal shape after evaporation	+50%	[23]
189	Water	*Luffa*	succussion strokes	0.8 × 10^−4^ g/mL	1	20	sm	0.05	100	Characteristics of crystal shape after evaporation	−20%	[23]
190	Water	*Luffa*	succussion strokes	0.8 × 10^−4^ g/mL	1	20	sm	0.05	10	Characteristics of crystal shape after evaporation	−20%	[23]
191	Water	*Spongia*	succussion strokes	0.8 × 10^−6^ g/mL	1	20	sm	0.05	100	Characteristics of crystal shape after evaporation	+5%	[23]
192	Water	*Spongia*	succussion strokes	0.8 × 10^−6^ g/mL	1	20	sm	0.05	10	Characteristics of crystal shape after evaporation	+10%	[23]
193	Water	IgG	Vertical shaking	IgG molecules 3 × 10^12^ cm^−3^	5	10	mm	20	30	DLS peak intensity ~200 nm (bubble count)	+300%	[6]
194	Water +36.7% ethanol	IgG	Vertical shaking	IgG molecules 3 × 10^12^ cm^−3^	5	10	mm	20	30	DLS peak intensity ~200 nm (bubble count)	+20%	[6]
195	Water	-	Shaking	-	30	5	mm	10	300	Concentration of molecular oxigen	−4%	[80]
196	Water	-	Shaking	-	30	5	mm	10	600	Concentration of molecular oxigen	−5%	[80]
197	Water	-	Shaking	-	30	5	mm	10	900	Concentration of molecular oxigen	−7%	[80]
198	Water	-	Shaking	-	30	5	mm	10	1800	Concentration of molecular oxigen	−8%	[80]
199	Water	-	Shaking	-	30	5	mm	10	2700	Concentration of molecular oxigen	−9%	[80]
200	Water	-	Shaking	-	30	5	mm	10	600	Water temperature	+2%	[80]
201	Water	-	Shaking	-	30	5	mm	10	900	Water temperature	+15%	[80]
202	Water	-	Shaking	-	30	5	mm	10	1800	Water temperature	+20%	[80]
203	Water	-	Shaking	-	30	5	mm	10	2700	Water temperature	+24%	[80]
204	Water	-	Shaking	-	15	5	mm	10	300	Concentration of molecular oxigen	−10%	[80]
205	Water	-	Shaking	-	45	5	mm	10	300	Concentration of molecular oxigen	−13%	[80]
206	Water	-	Shaking	-	60	5	mm	10	300	Concentration of molecular oxigen	−15%	[80]
207	Water	-	Shaking	-	30	5	mm	10	300	light scattering intensity	+50%	[80]
208	Water	-	Shaking	-	30	5	mm	10	900	pH	+12%	[80]
209	Water	-	Shaking	-	30	5	mm	10	1800	pH	+16%	[80]
210	Water	-	Shaking	-	30	5	mm	10	2700	pH	+17%	[80]
211	Water	-	Shaking	-	45	5	mm	10	300	pH	+6%	[80]
212	Water	-	Shaking	-	60	5	mm	10	300	pH	+8%	[80]
213	Water	-	Shaking	-	15	5	mm	10	300	Concentration of H_2_O_2_	+2000%	[80]
214	Water	-	Shaking	-	30	5	mm	10	300	Concentration of H_2_O_2_	+5000%	[80]
215	Water	-	Shaking	-	45	5	mm	10	300	Concentration of H_2_O_2_	+14000%	[80]
216	Water	-	Shaking	-	30	5	mm	10	60	Concentration of H_2_O_2_	+1000%	[80]
217	Water	-	Shaking	-	30	5	mm	10	150	Concentration of H_2_O_2_	+2500%	[80]
218	Water	-	Shaking	-	30	5	mm	10	210	Concentration of H_2_O_2_	+3500%	[80]
219	Water	-	Shaking	-	30	5	mm	10	300	Concentration of H_2_O_2_	+4500%	[80]
220	Water	-	Shaking	-	30	5	mm	10	180	Concentration of OH-radicals	+1500%	[80]
221	Water	-	Shaking	-	30	5	mm	10	300	Concentration of OH-radicals	+2500%	[80]
222	Water	-	Shaking	-	30	5	mm	10	420	Concentration of OH-radicals	+4000%	[80]
223	Water	-	Shaking	-	30	5	mm	10	600	Concentration of OH-radicals	+4300%	[80]
224	Water	-	Shaking	-	15	5	mm	10	300	Concentration of OH-radicals	+900%	[80]
225	Water	-	Shaking	-	45	5	mm	10	300	Concentration of OH-radicals	+5000%	[80]
226	Water	-	Shaking	-	60	5	mm	10	300	Concentration of OH-radicals	+9000%	[80]
227	Water	O_2_ gas	Creating an aerosol with N_2_	-	Constant	5	μL/min	0.011	60	Concentration of H_2_O_2_	+50	[90]
228	Water	O_2_ gas	Creating an aerosol with N_2_	-	Constant	5	μL/min	0.011	120	Concentration of H_2_O_2_	+50%	[90]
229	Water	O_2_ gas	Creating an aerosol with N_2_	-	Constant	5	μL/min	0.011	180	Concentration of H_2_O_2_	+100%	[90]
230	Water	O_2_ gas	Creating an aerosol with N_2_	-	Constant	5	μL/min	0.011	240	Concentration of H_2_O_2_	+500%	[90]
231	Water	O_2_ gas	Creating an aerosol with N_2_	-	Constant	5	μL/min	0.011	300	Concentration of H_2_O_2_	+600%	[90]
232	Water	O_2_ gas	Creating an aerosol with N_2_	-	Constant	5	μL/min	0.011	420	Concentration of H_2_O_2_	+1000%	[90]
233	Water	O_2_ gas	Creating an aerosol with N_2_	-	Constant	5	μL/min	0.011	1800	Concentration of H_2_O_2_	−15%	[90]
234	Water	O_2_ gas	Creating an aerosol with N_2_	-	Constant	5	μL/min	0.011	3600	Concentration of H_2_O_2_	−35%	[90]
235	Water	O_2_ gas	Creating an aerosol with N_2_	-	Constant	5	μL/min	0.011	14,400	Concentration of H_2_O_2_	−45%	[90]
236	Water	O_2_ gas	Creating an aerosol with N_2_	-	Constant	5	μL/min	0.011	21,600	Concentration of H_2_O_2_	−50%	[90]
237	Water	-	Mixing	-	12	1	sm	10	500	Absorption at 180–220 sm^−1^	+30%	[82]
238	Water	-	Mixing	-	12	1	sm	10	50	Wavelength of maximum absorption	+80% (280–340 нм)	[83]
239	Water	-	Mixing	-	12	1	sm	10	50	Wavelength of maximum absorption	+18% (280–330 нм)	[83]
240	Water	-	Mixing	-	12	1	sm	10	50	Fluorescence intensity	+400%	[83]
240	Water	-	Vibration		1,000,000	1	sm	10	500	Redox potentials Δ*E*	+1 mV (+100%)	[89]
240	Water coated by oil layes 10 mm		Vibration		1,000,000	1	sm	10	500	Redox potentials Δ*E*	+2.5 mV (+250%)	[89]
241	Water	AcEu solutions	Microfluidic mixing	-	Constant	400 35.5–250.6	Reynolds number	10	30	Position of the IR band maximum of the OH groups’ stretching vibrations on Raman spectrum	+0.1%	[94]
242	Water	AcEu solutions	Microfluidic mixing	-	Constant	400 35.5–250.6	Reynolds number	10	250	Position of the IR band maximum of the OH groups’ stretching vibrations on Raman spectrum	0.3	[94]
243	25 mM 4-(2-hydroxyethyl)-1-piperazineethanesulfonic acid sodium buffer (pH 7.0)	HEWL	Shaking	12.5 mg/mL	10	99	мкм	0.15	172,800	Protein crystallisation success	−25%	[92]
244	25 mM 4-(2-hydroxyethyl)-1-piperazineethanesulfonic acid sodium buffer (pH 7.0)	HEWL	Shaking	12.5 mg/mL	15	94.2	мкм	0.15	172,800	Protein crystallisation success	−25%	[92]
245	25 mM 4-(2-hydroxyethyl)-1-piperazineethanesulfonic acid sodium buffer (pH 7.0)	HEWL	Shaking	12.5 mg/mL	30	60	мкм	0.15	172,800	Protein crystallisation success	−25%	[92]
246	25 mM 4-(2-hydroxyethyl)-1-piperazineethanesulfonic acid sodium buffer (pH 7.0)	HEWL	Shaking	12.5 mg/mL	50	34.8	мкм	0.15	172,800	Protein crystallisation success	−25%	[92]
247	25 mM 4-(2-hydroxyethyl)-1-piperazineethanesulfonic acid sodium buffer (pH 7.0)	HEWL	Shaking	12.5 mg/mL	60	4	мкм	0.15	172,800	Protein crystallisation success	−25%	[92]
248	25 mM 4-(2-hydroxyethyl)-1-piperazineethanesulfonic acid sodium buffer (pH 7.0)	HEWL	Shaking	12.5 mg/mL	70	2.4	мкм	0.15	172,800	Protein crystallisation success	−25%	[92]
249	25 mM 4-(2-hydroxyethyl)-1-piperazineethanesulfonic acid sodium buffer (pH 7.0)	HEWL	Shaking	12.5 mg/mL	80	0.85	мкм	0.15	172,800	Protein crystallisation success	−25%	[92]
250	25 mM 4-(2-hydroxyethyl)-1-piperazineethanesulfonic acid sodium buffer (pH 7.0)	HEWL	Shaking	12.5 mg/mL	90	0.6	мкм	0.15	172,800	Protein crystallisation success	−25%	[92]
251	25 mM 4-(2-hydroxyethyl)-1-piperazineethanesulfonic acid sodium buffer (pH 7.0)	HEWL	Shaking	12.5 mg/mL	100	0.4	мкм	0.15	172,800	Protein crystallisation success	−25%	[92]
252	0.1 M acetate buffer pH 4.5	HEWL	Ultrasound	25 mg/mL	100,000	100,000	mW/cm^2^	9.4	40	Number of protein crystals	+150%	[93]
253	0.1 M acetate buffer pH 4.5	HEWL	Ultrasound	25 mg/mL	100,000	100,000	mW/cm^2^	9.4	70	Number of protein crystals	+600%	[93]
254	0.1 M acetate buffer pH 4.8	HEWL 40,000 U/mg	Flow rate oscillations	50 mg/mL	1.6	20	mm	0.5	3600	Concentration of protein after precipitation in 100 mg/mL NaCl	−5%	[209]
255	0.1 M acetate buffer pH 4.8	HEWL 40,000 U/mg	Flow rate oscillations	50 mg/mL	2.5	20	mm	0.5	3600	Concentration of protein after precipitation in 100 mg/mL NaCl	−10%	[209]
256	0.1 M acetate buffer pH 4.8	HEWL 40,000 U/mg	Flow rate oscillations	50 mg/mL	3.3	20	mm	0.5	3600	Concentration of protein after precipitation in 100 mg/mL NaCl	−15%	[209]
257	0.1 M acetate buffer pH 4.8	HEWL 40,000 U/mg	Flow rate oscillations	50 mg/mL	1.6	20	mm	0.5	4800	Concentration of protein after precipitation in 100 mg/mL NaCl	−10%	[209]
258	0.1 M acetate buffer pH 4.8	HEWL 40,000 U/mg	Flow rate oscillations	50 mg/mL	2.5	20	mm	0.5	4800	Concentration of protein after precipitation in 100 mg/mL NaCl	−20%	[209]
259	0.1 M acetate buffer pH 4.8	HEWL 40,000 U/mg	Flow rate oscillations	50 mg/mL	3.3	20	mm	0.5	4800	Concentration of protein after precipitation in 100 mg/mL NaCl	−40%	[209]
260	0.1 M acetate buffer pH 4.8	HEWL 40,000 U/mg	Flow rate oscillations	50 mg/mL	1.6	20	mm	0.5	10,800	Concentration of protein after precipitation in 100 mg/mL NaCl	−35%	[209]
261	0.1 M acetate buffer pH 4.8	HEWL 40,000 U/mg	Flow rate oscillations	50 mg/mL	2.5	20	mm	0.5	10,800	Concentration of protein after precipitation in 100 mg/mL NaCl	−40%	[209]
262	0.1 M acetate buffer pH 4.8	HEWL 40,000 U/mg	Flow rate oscillations	50 mg/mL	3.3	20	mm	0.5	10,800	Concentration of protein after precipitation in 100 mg/mL NaCl	−50%	[209]
263	0.1 M acetate buffer pH 4.8	HEWL 40,000 U/mg	Flow rate oscillations	90 mg/mL	1.6	20	mm	0.5	1800	Concentration of protein after precipitation in 100 mg/mL NaCl	−5%	[209]
264	0.1 M acetate buffer pH 4.8	HEWL 40,000 U/mg	Flow rate oscillations	90 mg/mL	2.5	20	mm	0.5	1800	Concentration of protein after precipitation in 100 mg/mL NaCl	−5%	[209]
265	0.1 M acetate buffer pH 4.8	HEWL 40,000 U/mg	Flow rate oscillations	90 mg/mL	3.3	20	mm	0.5	1800	Concentration of protein after precipitation in 100 mg/mL NaCl	−60%	[209]
266	0.1 M acetate buffer pH 4.8	HEWL 40,000 U/mg	Flow rate oscillations	90 mg/mL	1.6	20	mm	0.5	3600	Concentration of protein after precipitation in 100 mg/mL NaCl	−50%	[209]
267	0.1 M acetate buffer pH 4.8	HEWL 40,000 U/mg	Flow rate oscillations	90 mg/mL	2.5	20	mm	0.5	3600	Concentration of protein after precipitation in 100 mg/mL NaCl	−70%	[209]
268	0.1 M acetate buffer pH 4.8	HEWL 40,000 U/mg	Flow rate oscillations	90 mg/mL	3.3	20	mm	0.5	3600	Concentration of protein after precipitation in 100 mg/mL NaCl	−80%	[209]
269	0.1 M acetate buffer pH 4.8	HEWL 40,000 U/mg	Flow rate oscillations	90 mg/mL	1.6	20	mm	0.5	5400	Concentration of protein after precipitation in 100 mg/mL NaCl	−75%	[209]
270	0.1 M acetate buffer pH 4.8	HEWL 40,000 U/mg	Flow rate oscillations	90 mg/mL	2.5	20	mm	0.5	5400	Concentration of protein after precipitation in 100 mg/mL NaCl	−78%	[209]
271	0.1 M acetate buffer pH 4.8	HEWL 40,000 U/mg	Flow rate oscillations	90 mg/mL	3.3	20	mm	0.5	5400	Concentration of protein after precipitation in 100 mg/mL NaCl	−80%	[209]
272	0.1 M acetate buffer pH 4.8	HEWL 40,000 U/mg	Flow rate oscillations	50 mg/mL	0.1	20	mm	0.5	5400	Concentration of protein after precipitation in 100 mg/mL NaCl	−5%	[209]
273	0.1 M acetate buffer pH 4.8	HEWL 40,000 U/mg	Flow rate oscillations	50 mg/mL	0.2	20	mm	0.5	5400	Concentration of protein after precipitation in 100 mg/mL NaCl	−10%	[209]
274	0.1 M acetate buffer pH 4.8	HEWL 40,000 U/mg	Flow rate oscillations	50 mg/mL	0.5	20	mm	0.5	5400	Concentration of protein after precipitation in 100 mg/mL NaCl	−45%	[209]
275	0.1 M acetate buffer pH 4.8	HEWL 40,000 U/mg	Flow rate oscillations	50 mg/mL	1	20	mm	0.5	5400	Concentration of protein after precipitation in 100 mg/mL NaCl	−75%	[209]
276	0.1 M acetate buffer pH 4.8	HEWL 40,000 U/mg	Flow rate oscillations	50 mg/mL	0.5	5	mm	0.5	5400	Concentration of protein after precipitation in 100 mg/mL NaCl	−10%	[209]
277	0.1 M acetate buffer pH 4.8	HEWL 40,000 U/mg	Flow rate oscillations	50 mg/mL	0.5	10	mm	0.5	5400	Concentration of protein after precipitation in 100 mg/mL NaCl	−20%	[209]
278	0.1 M acetate buffer pH 4.8	HEWL 40,000 U/mg	Flow rate oscillations	50 mg/mL	0.5	30	mm	0.5	5400	Concentration of protein after precipitation in 100 mg/mL NaCl	−80%	[209]
279	Water	1 mmol (110 μL) benzaldehyde was with 1 mmol (145 μL) aminoacetaldehyde diethyl	Atomization (formations of nanobubbles in the flow N_2_)	1 mmol	0	30	mL/min	0.00000002	0.01	Pomeranz–Fritsch reaction rate	+588%	[29]
280	Methanol	1 mmol (110 μL) benzaldehyde was with 1 mmol (145 μL) aminoacetaldehyde diethyl	Atomization (formations of nanobubbles in the flow N_2_)	1 mmol	0	30	mL/min	0.00000002	0.01	Pomeranz–Fritsch reaction rate	+644%	[29]
281	ACN/DMF (1:1; *v*/*v*)	1 mmol (110 μL) benzaldehyde was with 1 mmol (145 μL) aminoacetaldehyde diethyl	Atomization (formations of nanobubbles in the flow N_2_)	1 mmol	0	30	mL/min	0.00000002	0.01	Pomeranz–Fritsch reaction rate	+233%	[29]
282	1% (*v*/*v*) m-NBA in water	1 mmol (110 μL) benzaldehyde was with 1 mmol (145 μL) aminoacetaldehyde diethyl	Atomization (formations of nanobubbles in the flow N_2_)	1 mmol	0	30	mL/min	0.00000002	0.01	Pomeranz–Fritsch reaction rate	+736%	[29]
283	Water	Bi_3_TiNbO_9_	Ultrasound	not applicable. solid catalyst	40,000	50,000	mW/cmm^2^	10	18,000	H_2_O_2_ production	+40,705%	[126]
284	Water	-	Shaking	-	30	1	sm	10	60	H_2_O_2_ production	+100%	[84]
285	Water	-	Shaking	-	30	1	sm	10	150	H_2_O_2_ production	+200%	[84]
286	Water	-	Shaking	-	30	1	sm	10	210	H_2_O_2_ production	+280%	[84]
287	Water	-	Shaking	-	30	1	sm	10	300	H_2_O_2_ production	+300%	[84]
288	Water	-	Shaking	-	30	0.5	sm	10	60	H_2_O_2_ production	+20%	[86]
289	Water	-	Shaking	-	30	0.5	sm	10	150	H_2_O_2_ production	+50%	[86]
290	Water	-	Shaking	-	30	0.5	sm	10	210	H_2_O_2_ production	+60%	[86]
291	Water	-	Shaking	-	30	0.5	sm	10	300	H_2_O_2_ production	+80%	[86]
292	Water	-	Shaking (turbulent)	-	15	1	sm	10	300	H_2_O_2_ production	+150%	[85]
293	Water	-	Shaking (turbulent)	-	30	1	sm	10	300	H_2_O_2_ production	+250%	[85]
294	Water	-	Shaking (turbulent)	-	60	1	sm	10	300	H_2_O_2_ production	+1500%	[85]
295	Water	-	Shaking (turbulent)	-	30	1	sm	10	900	[O_2_] concentration	+100%	[85]
296	Water	-	Shaking (turbulent)	-	30	1	sm	10	1500	[O_2_] concentration	+100%	[85]
297	Water	-	Shaking (turbulent)	-	30	1	sm	10	1800	[O_2_] concentration	+150%	[85]
298	Water	-	Shaking (turbulent)	-	30	1	sm	10	2400	[O_2_] concentration	+160%	[85]
299	Water	-	Shaking (turbulent)	-	30	1	sm	10	2700	[O_2_] concentration	+200%	[85]
300	Water	-	Shaking (turbulent)	-	30	1	sm	10	4200	[O_2_] concentration	+200%	[85]
301	Water	-	Shaking (laminar)	-	30	1	sm	10	900	[O_2_] concentration	+10%	[85]
302	Water	-	Shaking (laminar)	-	30	1	sm	10	1500	[O_2_] concentration	+10%	[85]
303	Water	-	Shaking (laminar)	-	30	1	sm	10	1800	[O_2_] concentration	+20%	[85]
304	Water	-	Shaking (laminar)	-	30	1	sm	10	2400	[O_2_] concentration	+20%	[85]
305	Water	-	Shaking (laminar)	-	30	1	sm	10	2700	[O_2_] concentration	+25%	[85]
306	Water	-	Shaking (laminar)	-	30	1	sm	10	4200	[O_2_] concentration	+25%	[85]
307	*Hedysarum laeve* seedlings	-	Shaking 45°	-	2	6.4	m/sm^2^	1	60	Primary stem length	−15%	[55]
308	*Hedysarum laeve* seedlings	-	Shaking 45°	-	2	6.4	m/sm^2^	1	60	Leaf numbe	−15%	[55]
309	*Hedysarum laeve* seedlings	-	Shaking 45°	-	2	6.4	m/sm^2^	1	60	Basal diameter	−12%	[55]
310	*Hedysarum laeve* seedlings	-	Shaking 45°	-	2	6.4	m/sm^2^	1	60	Total biomass	−30%	[55]
311	Water	-	Downfall of drops	0	0	1	m	0.05	0.1	[H_2_O_2_] concentration	+300%	[27]
312	Water	-	Downfall of drops	0	0	2	m	0.05	0.1	[H_2_O_2_] concentration	+550%	[27]
313	Water	-	Downfall of drops	0	0	3	m	0.05	0.1	[H_2_O_2_] concentration	+750%	[27]
314	Water	-	Downfall of drops	0	0	4	m	0.05	0.1	[H_2_O_2_] concentration	+900%	[27]
315	Water	-	Downfall of drops	0	0	1	m	0.05	0.1	[OH] concentration	+200%	[27]
316	Water	-	Downfall of drops	0	0	2	m	0.05	0.1	[OH] concentration	+350%	[27]
317	Water	-	Downfall of drops	0	0	3	m	0.05	0.1	[OH] concentration	+500%	[27]
318	Water	-	Downfall of drops	0	0	4	m	0.05	0.1	[OH] concentration	+550%	[27]
319	Water	PNaAMPS/PAAm double-network (DN) hydrogels	Stretching	1.4 М	0	1.07	Value of the first Lame parameter λ	20	1800	Normalised width w(t)/w(0)	+2%	[95]
321	Water	PNaAMPS/PAAm double-network (DN) hydrogels	Stretching	1.4 М	0	1.14	Value of the first Lame parameter λ	20	1800	Normalised width w(t)/w(0)	+2%	[95]
322	Water	PNaAMPS/PAAm double-network (DN) hydrogels	Stretching	1.4 М	0	1.21	Value of the first Lame parameter λ	20	1800	Normalised width w(t)/w(0)	+1%	[95]
323	Water	PNaAMPS/PAAm double-network (DN) hydrogels	Stretching	1.4 М	0	1.23	Value of the first Lame parameter λ	20	1800	Normalised width w(t)/w(0)	+0%	[95]
324	Water	PNaAMPS/PAAm double-network (DN) hydrogels	Stretching	1.4 М	0	1.27	Value of the first Lame parameter λ	20	1800	Normalised width w(t)/w(0)	−1.5%	[95]
325	Water	PNaAMPS/PAAm double-network (DN) hydrogels	Stretching	1.4 М	0	1.32	Value of the first Lame parameter λ	20	1800	Normalised width w(t)/w(0)	−2%	[95]
326	Water	PNaAMPS/PAAm double-network (DN) hydrogels	Stretching	1.4 М	0	1.38	Value of the first Lame parameter λ	20	1800	Normalised width w(t)/w(0)	−3%	[95]
327	Water	PNaAMPS/PAAm double-network (DN) hydrogels	Stretching	1.4 М	0	1.07	Value of the first Lame parameter λ	20	1800	normalized engineering stress σ(t)/σ(0)	−6%	[95]
328	Water	PNaAMPS/PAAm double-network (DN) hydrogels	Stretching	1.4 М	0	1.14	Value of the first Lame parameter λ	20	1800	normalized engineering stress σ(t)/σ(0)	−0%	[95]
329	Water	PNaAMPS/PAAm double-network (DN) hydrogels	Stretching	1.4 М	0	1.21	Value of the first Lame parameter λ	20	1800	normalized engineering stress σ(t)/σ(0)	−0%	[95]
330	Water	PNaAMPS/PAAm double-network (DN) hydrogels	Stretching	1.4 М	0	1.23	Value of the first Lame parameter λ	20	1800	normalized engineering stress σ(t)/σ(0)	−1%	[95]
331	Water	PNaAMPS/PAAm double-network (DN) hydrogels	Stretching	1.4 М	0	1.27	Value of the first Lame parameter λ	20	1800	normalized engineering stress σ(t)/σ(0)	−3%	[95]
332	Water	PNaAMPS/PAAm double-network (DN) hydrogels	Stretching	1.4 М	0	1.32	Value of the first Lame parameter λ	20	1800	normalized engineering stress σ(t)/σ(0)	−14%	[95]
333	Water	PNaAMPS/PAAm double-network (DN) hydrogels	Stretching	1.4 М	0	1.38	Value of the first Lame parameter λ	20	1800	normalized engineering stress σ(t)/σ(0)	−17%	[95]
334	waste water after blowing by 99.99% N_2_	*T. denitrificans.* immobilised on electrod	Mixing	99.99% N_2_	10	5	mm	26.2	259,200	Concntration of Na+	−20%	[79]
335	waste water after blowing by 99.99% N_2_	*T. denitrificans.* immobilised on electrod	Mixing	99.99% N_2_	10	5	mm	26.2	259,200	Electrode voltage	+80%	[79]
336	waste water after blowing by 99.99% N_2_	*T. denitrificans.* immobilised on electrod	Mixing	99.99% N_2_	10	5	mm	26.2	259,200	Consumption of NO_3_^-^	+35%	[79]
337	Water	*-*	Mixing	-	10	37	mm	30	180	Concentration of bulk nanobubbles	+150%	[96]
338	Water	*-*	Mixing	-	5	37	mm	30	180	Concentration of bulk nanobubbles	+80%	[96]
339	Water	*-*	Mixing	-	15	37	mm	30	180	Concentration of bulk nanobubbles	+200%	[96]
340	Water	*-*	Mixing	-	15	37	mm	30	180	Concentration of bulk nanobubbles	+30%	[96]
341	Water	*-*	Mixing	-	15	37	mm	30	60	Concentration of bulk nanobubbles	+200%	[96]
342	Water	*-*	Mixing	-	15	37	mm	30	360	Concentration of bulk nanobubbles	+1500%	[96]
343	Water	*-*	Mixing	-	15	37	mm	30	600	Concentration of bulk nanobubbles	+1100%	[96]
344	Water	*-*	Ultrasound	-	22,000	2000	mW/cm^2^	5000	300	pH	+6%	[121]
345	Water	*-*	Ultrasound	-	100,0001,000,000	2000	mW/cm^2^	5000	300	pH	−17%	[121]
346	Water (artesian)	total hardness. carbonate hardness; Ca^+2^ content; Mg^+2^ content; pH 7. sulfates. chlorides	Ultrasound	16.6 mg-eq/L. 4.8 mg-eq/. 17.7 mg/L. 8.9 mg/L. 7.8 mg/L. 576 mg/L. 12.0 mg/L	22,000	2000	mW/cm^2^	5000	300	pH	+12%	[121]
347	Water	Clay particles	Ultrasound	20 mg/mL	20,000	2000	mW/cm^2^	1000	10	Precipitation rate	−30%	[124]
348	Water	Clay particles	Ultrasound	20 mg/mL	20,000	2000	mW/cm^2^	1000	30	Precipitation rate	+1%	[124]
349	Water	Clay particles	Ultrasound	20 mg/mL	20,000	2000	mW/cm^2^	1000	60	Precipitation rate	+50%	[124]
350	Water	Clay particles	Ultrasound	20 mg/mL	20,000	2000	mW/cm^2^	1000	120	Precipitation rate	+60%	[124]
351	Water	Clay particles	Ultrasound	20 mg/mL	20,000	2000	mW/cm^2^	1000	300	Precipitation rate	+101%	[124]
352	Mouse	in vivo	Sinusoidal vibrations	-	45	0.3	g	21	37,800	Number of lectin-positive vessels	−29%	[20]
353	Mouse	in vivo	Sinusoidal vibrations	-	45	0.3	g	21	37,800	α-actin-positive vessels	−36%	[20]
355	Rabbit	in vivo	Shaking	-	60	5	g	4900	2,160,000	Myelin fibre diameter	+80%	[53]
356	Human	in vivo	Vibration	-	25	3	m/s^2^	92,900	60	vibration intensity transmission	+90%	[51]
357	Human	in vivo	Vibration	-	200	3	m/s^2^	92,900	60	vibration intensity transmission	+900%	[51]
358	Human	in vivo	Vibration	-	25	3	m/s^2^	92,900	60	Apparent mass	−50%	[51]
359	Human	in vivo	Vibration	-	200	3	m/s^2^	92,900	60	Apparent mass	−80%	[51]
360	Human	in vivo	Vibration	-	25	3	m/s^2^	92,900	60	Mechanical impedance	+25%	[51]
361	Human	in vivo	Vibration	-	200	3	m/s^2^	92,900	60	Mechanical impedance	+150%	[51]
362	Rat	in vivo	Vibration	-	80	32	m/s^2^	205	90,000	Length of nerve regeneration by 6 days	+57%	[54]
363	Rat	in vivo	Vibration	-	80	32	m/s^2^	205	90,000	Length of nerve regeneration by 6 days	+23%	[54]
364	Rat	in vivo	Vibration	-	81	0.5	mm	240	28,800	Expression of IGF-I in nerve fibers	+100%	[210]
365	Rat. tail	in vivo	Vertical shaking	-	62.5	49	m/s^2^	6	144,00	Expression of *MT-1a*	+700%	[40]
366	Rat. tail	in vivo	Vertical shaking	-	125	49	m/s^2^	6	144,00	Expression of *MT-1a*	+660%	[40]
367	Rat. tail	in vivo	Vertical shaking	-	250	49	m/s^2^	6	144,00	Expression of *MT-1a*	+630%	[40]
368	Rat. tail	in vivo	Vertical shaking	-	62.5	49	m/s^2^	6	144,00	Expression of *IL-6*	+900%	[40]
369	Rat. tail	in vivo	Vertical shaking	-	125	49	m/s^2^	6	144,00	Expression of *IL-6*	+500%	[40]
370	Rat. tail	in vivo	Vertical shaking	-	250	49	m/s^2^	6	14,400	Expression of *IL-6*	+300%	[40]
371	Rat. tail	in vivo	Vertical shaking	-	62.5	49	m/s^2^	6	144,00	Expression of *IL-6*	+400%	[40]
372	Rat. tail	in vivo	Vertical shaking	-	125	49	m/s^2^	6	144,00	Expression of *IL-6*	+220%	[40]
373	Rat. tail	in vivo	Vertical shaking	-	250	49	m/s^2^	6	14,400	Internal diameter of the caudal vein	−70%	[40]
374	Rat. tail	in vivo	Vertical shaking	-	250	49	m/s^2^	6	144,00	Vascular smooth muscle thickness	+35%	[40]
375	Rat. tail	in vivo	Vertical shaking	-	125	49	m/s^2^	6	14,400	Synthesis of nitrotyrosine	+125%	[40]
376	Rat. tail	in vivo	Vertical shaking	-	250	49	m/s^2^	6	144,00	Synthesis of nitrotyrosine	+150%	[40]
377	Rat. tail	in vivo	Vertical shaking	-	125	49	m/s^2^	6	144,00	Synthesis of IL-6	+50%	[40]
378	Rat. tail	in vivo	Vertical shaking	-	250	49	m/s^2^	6	14,400	Synthesis of IL-6	+70%	[40]
379	Rat. tail	in vivo	Vertical shaking	-	60	49	m/s^2^	5	14,000	Number of vacuoles in endothelial cells	+2900%	[41]
380	Rat. tail	in vivo	Vertical shaking	-	120	49	m/s^2^	5	14,000	Number of vacuoles in endothelial cells	+1300%	[41]
381	Rat. tail	in vivo	Vertical shaking	-	30	49	m/s^2^	5	144,00	Number of ruptures of the inner vessel wall	+1600:	[41]
382	Rat. tail	in vivo	Vertical shaking	-	69	49	m/s^2^	5	144,00	Number of ruptures of the inner vessel wall	+2100%	[41]
383	Rat. tail	in vivo	Vertical shaking	-	120	49	m/s^2^	5	14,400	Number of ruptures of the inner vessel wall	+1500%	[41]
384	Rat. tail	in vivo	Vertical shaking	-	800	49	m/s^2^	5	14,400	Number of ruptures of the inner vessel wall	+1600%	[41]
385	Rat. tail	in vivo	Vertical shaking	-	30	49	m/s^2^	5	14,400	Expression of NFATc3	+100%	[41]
386	Rat. tail	in vivo	Vertical shaking	-	60	49	m/s^2^	5	14,400	Expression of NFATc3	+200%	[41]
387	Rat. tail	in vivo	Vertical shaking	-	120	49	m/s^2^	5	14,400	Expression of NFATc3	+200%	[41]
388	Rat. lower limbs	in vivo	Vertical shaking	-	30	49	m/s^2^	15	57,600	Total plasma creatine phosphokinase (t-CPK) activity	+1830%	[42]
389	Rat. lower limbs	in vivo	Vertical shaking	-	60	49	m/s^2^	15	57,600	Total plasma creatine phosphokinase (t-CPK) activity	+760%	[42]
390	Rat. lower limbs	in vivo	Vertical shaking	-	120	49	m/s^2^	15	57,600	Total plasma creatine phosphokinase (t-CPK) activity	+700%	[42]
391	Rat. lower limbs	in vivo	Vertical shaking	-	240	49	m/s^2^	15	57,600	Total plasma creatine phosphokinase (t-CPK) activity	+400%	[42]
392	Rat. lower limbs	in vivo	Vertical shaking	-	480	49	m/s^2^	15	57,600	Total plasma creatine phosphokinase (t-CPK) activity	+200%	[42]
393	Culture medium DMEM	Mouse Fibroblast Cells	Sinusoidal shaking	-	1250	16.5	m/s^2^	5	900	Cells viability	−59%	[57]
394	Culture medium DMEM	Mouse Fibroblast Cells	Shock shaking	-	1250	12.9	m/s^2^	5	900	Cells viability	−99%	[57]
395	Culture medium DMEM	Mouse red blood cells	Shock shaking	-	1250	30,000	m/s^2^	5	900	Erythrocyte lysis	+100%	[57]
396	Culture medium DMEM	Mouse red blood cells	Shock shaking	-	1250	15,000	m/s^2^	5	900	Erythrocyte lysis	+0.4%	[57]
397	Culture medium DMEM	Mouse red blood cells	Shock shaking	-	1250	2000	m/s^2^	5	900	Erythrocyte lysis	+0.07%	[57]
398	Culture medium DMEM	Mouse red blood cells	Shock shaking	-	1250	1000	m/s^2^	5	900	Erythrocyte lysis	+0.1%	[57]
399	Culture medium DMEM	Mouse red blood cells	Shock shaking	-	1250	500	m/s^2^	5	900	Erythrocyte lysis	+0.1%	[57]
400	Human	in vivo	Vibration	-	5.5	74.4	N	60,000	600	Peak inspiratory flow rate	+136%	[52]
401	Human	in vivo	Vibration	-	5.5	74.4	N	60,000	600	Inspired volume	+200%	[52]
402	Human	in vivo	Vibration	-	5.5	74.4	N	60,000	600	expired volume	+290%	[52]
403	Human. healthy pre-pubertal boys	in vivo	Vibration (platform)	-	40	2.1	g	34,000	600	Legs’ skin temperature	+8%	[46]
404	Human. healthy pre-pubertal boys	in vivo	Vibration (platform)	-	40	2.1	g	34,000	3000	P1NP plasma level after 8 days	+18%	[46]
405	Human. healthy pre-pubertal boys	in vivo	Vibration (platform)	-	40	2.1	g	34,000	3000	CTx plasma level after 8 days	+10%	[46]
406	Rat with titanium hip implants	in vivo	Vibration (whole body)	-	80	0.3	g	353	300	Bone-to-implant contact	+63%	[48]
407	Rat with titanium hip implants	in vivo	Vibration (whole body)	-	140	0.3	g	353	300	Bone-to-implant contact	+65%	[48]
408	Rat with titanium hip implants	in vivo	Vibration (whole body)	-	26	0.3	g	353	300	Peri-implant bone formation	+20%	[48]
409	Rat with titanium hip implants	in vivo	Vibration (whole body)	-	80	0.3	g	353	300	Peri-implant bone formation	+25%	[48]
410	Rat with titanium hip implants	in vivo	Vibration (whole body)	-	140	0.043	g	353	300	Peri-implant bone formation	+19%	[48]
411	Rat with titanium hip implants	in vivo	Vibration (whole body)	-	140	0.3	g	353	300	Peri-implant bone formation	+25%	[48]
412	Rat with removed ovaries (osteoporosis model)	in vivo	Vibration (whole body)	-	30	0.3	g	285	134,400	Obesity	−50%	[28]
413	Rat with removed ovaries (osteoporosis model)	in vivo	Vibration (whole body)		30	0.3	g	285	67,200	Bone mineral densities	+15%	[28]
414	Rat with removed ovaries (osteoporosis model)	in vivo	Vibration (whole body)	-	30	0.3	g	285	134,400	Vertebra compression test (maximum load)	−18%	[28]
415	Rat (healthy)	in vivo	Vibration (whole body)	-	30	0.3	g	285	134,400	vertebra compression test (rigidity)	−45%	[28]
416	Rat with removed ovaries (osteoporosis model)	in vivo	Vibration (whole body)	-	30	0.3	g	285	134,400	Osteoblastic Cell Viability	−10%	[28]
417	Rat (healthy)	in vivo	Vibration (whole body)	-	30	0.3	g	285	134,400	Osteoblastic Cell Viability	−12%	[28]
418	Rat (healthy)	in vivo	Vibration (whole body)	-	30	0.3	g	285	134,400	Alkaline phosphatase (ALP) activity 4 day	+15%	[28]
419	Rat (healthy)	in vivo	Vibration (whole body)	-	30	0.3	g	285	134,400	alkaline phosphatase (ALP) activity 7 day	−10%	[28]
420	Rat (healthy)	in vivo	Vibration (whole body)	-	30	0.3	g	285	134,400	Expression of *ALP*	−66%	[28]
421	Rat with removed ovaries (osteoporosis model)	in vivo	Vibration (whole body)	-	30	0.3	g	285	134,400	Expression of *ALP*	−50%	[28]
422	Rat (healthy)	in vivo	Vibration (whole body)	-	30	0.3	g	285	134,400	Expression of *MMP2*	−40%	[28]
423	Rat with removed ovaries (osteoporosis model)	in vivo	Vibration (whole body)	-	30	0.3	g	285	134,400	Expression of *MMP2*	−40%	[28]
424	Rat (healthy)	in vivo	Vibration (whole body)	-	30	0.3	g	285	134,400	Expression of *OSX*	−10%	[28]
425	Rat with removed ovaries (osteoporosis model)	in vivo	Vibration (whole body)	-	30	0.3	g	285	134,400	Expression of *OSX*	+20%	[28]
426	C57BL/6 mouse	in vivo	Vibration (whole body)	-	90	0.75	m/s^2^	27.5	300	Arterial blood pressure	+25%	[39]
427	C57BL/6 mouse	in vivo	Vibration (whole body)	-	80	0.75	m/s^2^	27.5	300	Heart beat rate	+19%	[39]
428	C57BL/6 mouse	in vivo	Vibration (whole body)	-	90	0.75	m/s^2^	27.5	300	Heart beat rate	+17%	[39]
429	Human	in vivo	Vibration (whole body)	-	30	0.3	m/s^2^	61500	14,400	Bone mineralisation density	+40%	[44]
430	Human	in vivo	Vibration (whole body)	-	30	0.3	m/s^2^	61500	14,400	TRAP synthesis	−20%	[44]
431	Culture medium DMEM	Human embryonic stem cells (hESC)	Ultrasound	-	19,690,000	24,000	mW	5	7200	Expression of markers of neuronal differentiation and *Alk*	+250%	[116]
432	Culture medium DMEM	Human embryonic stem cells (hESC)	Ultrasound	-	19,690,000	310,000	mW	5	7200	Expression of markers of neuronal differentiation and *Alk*	+200%	[116]
433	Culture medium DMEM	Human embryonic stem cells (hESC)	Ultrasound	-	19,690,000	24,000	mW	5	7200	Expression of *Cenpf*	+40%	[116]
434	Culture medium DMEM	Human embryonic stem cells (hESC)	Ultrasound	-	19,690,000	31,000	mW	5	7200	Expression of *Cenpf*	+20%	[116]
435	Culture medium DMEM	Human embryonic stem cells (hESC)	Ultrasound	-	19,690,000	39,000	mW	5	7200	Expression of *Cenpf*	−30%	[116]
436	Culture medium DMEM	Human embryonic stem cells (hESC)	Ultrasound	-	19,690,000	24,000	mW	5	7200	Expression of actin	−30%	[116]
437	Culture medium DMEM	Human embryonic stem cells (hESC)	Ultrasound	-	19,690,000	31,000	mW	5	7200	Expression of actin	−40%	[116]
438	Culture medium DMEM	Human embryonic stem cells (hESC)	Ultrasound	-	19,690,000	39,000	mW	5	7200	Expression of actin	−80%	[116]
439	Culture medium DMEM	Human embryonic stem cells (hESC)	Ultrasound	-	19,690,000	24,000	mW	5	7200	Expression of *Pcdh17*	+60%	[116]
440	Culture medium DMEM	Human embryonic stem cells (hESC)	Ultrasound	-	19,690,000	31,000	mW	5	7200	Expression of *Pcdh17*	+70%	[116]
441	Culture medium DMEM	Human embryonic stem cells (hESC)	Ultrasound	-	19,690,000	39,000	mW	5	7200	Expression of *Pcdh17*	+20%	[116]
442	Culture medium DMEM	SHED: stem cells from human exfoliated deciduous teeth	Centrifugation	-	-	100	g	5	30 min. every 24 h.. 7 дней	Cells proliferation	−16.32%	[25]
443	Culture medium DMEM	SHED: stem cells from human exfoliated deciduous teeth	Centrifugation	-	-	200	g	5	30 min. every 24 h.. 7 дней	Cells proliferation	−18.36%	[25]
444	Culture medium DMEM	SHED: stem cells from human exfoliated deciduous teeth	Centrifugation	-	-	300	g	5	30 min. every 24 h.. 7 дней	Cells proliferation	−16.32%	[25]
445	Culture medium α-MEM	MC3T3-E1	Vibration	-	12.5	0.5	G	5	1,382,400	Confluency	+19.4%	[26]
446	Culture medium α-MEM	MC3T3-E1	Vibration	-	12.5	0.5	G	4	1,123,200	Confluency	+15.6%	[26]
447	Culture medium α-MEM	MC3T3-E1	Constant flow	-	0	0.28 ± 0.02	mL/min	4	1,382,400	Confluency	−22.2%	[26]
448	Culture medium α-MEM	MC3T3-E1	Constant flow	-	0	0.28 ± 0.02	mL/min	4	1,123,200	Confluency	+12.5%	[26]
449	Culture medium α-MEM	human PDL stem cells	Vibration	-	50	0.3	G	10	30 min. every 24 h.	Alkaline phosphatase activity	+123.8%	[211]
450	Culture medium α-MEM	human PDL stem cells	Vibration	-	60	0.3	G	10	30 min. every 24 h.	Alkaline phosphatase activity	+96.23%	[211]
451	Culture medium α-MEM	human PDL stem cells	Vibration	-	10	0.3	G	10	30 min. every 24 h.	Expression of gene *Runx2*	−43.75%	[211]
452	Culture medium α-MEM	human PDL stem cells	Vibration	-	40	0.3	G	10	30 min. every 24 h.	Expression of gene *Runx2*	+175%	[211]
453	Culture medium α-MEM	human PDL stem cells	Vibration	-	50	0.3	G	10	30 min. every 24 h.	Expression of gene *Runx2*	+275%	[211]
454	Culture medium α-MEM	human PDL stem cells	Vibration	-	60	0.3	G	10	30 min. every 24 h.	Expression of gene *Runx2*	+68.75%	[211]
455	Culture medium α-MEM	human PDL stem cells	Vibration	-	90	0.3	G	10	30 min. every 24 h.	Expression of gene *Runx2*	+34.37%	[211]
456	Culture medium α-MEM	human PDL stem cells	Vibration	-	180	0.3	G	10	30 min. every 24 h.	Expression of gene *Runx2*	−25%	[211]
457	Culture medium α-MEM	human PDL stem cells	Vibration	-	20	0.3	G	10	30 min. every 24 h.	Expression of gene *Osx*	−50%	[211]
458	Culture medium α-MEM	human PDL stem cells	Vibration	-	40	0.3	G	10	30 min. every 24 h.	Expression of gene *Osx*	+62.5%	[211]
459	Culture medium α-MEM	human PDL stem cells	Vibration	-	50	0.3	G	10	30 min. every 24 h.	Expression of gene *Osx*	+109.3%	[211]
460	Culture medium α-MEM	human PDL stem cells	Vibration	-	60	0.3	G	10	30 min. every 24 h.	Expression of gene *Osx*	−43.75%	[211]
461	Culture medium α-MEM	human PDL stem cells	Vibration	-	120	0.3	G	10	30 min. every 24 h.	Expression of gene *Osx*	−43.75%	[211]
462	Culture medium α-MEM	human PDL stem cells	Vibration	-	150	0.3	G	10	30 min. every 24 h.	Expression of gene *Osx*	−56.25%	[211]
463	Culture medium α-MEM	human PDL stem cells	Vibration	-	180	0.3	G	10	30 min. every 24 h.	Expression of gene *Osx*	+37.5%	[211]
464	Culture medium α-MEM	human PDL stem cells	Vibration	-	40	0.3	G	10	30 min. every 24 h.	Osteocalcin levels (OCN)	+340.9%	[211]
465	Culture medium α-MEM	human PDL stem cells	Vibration	-	50	0.3	G	10	30 min. every 24 h.	Osteocalcin levels (OCN)	+390.9%	[211]
466	Culture medium α-MEM	human PDL stem cells	Vibration	-	60	0.3	G	10	30 min. every 24 h.	Osteocalcin levels (OCN)	+318.18%	[211]
467	Culture medium α-MEM	human PDL stem cells	Vibration	-	90	0.3	G	10	30 min. every 24 h.	Osteocalcin levels (OCN)	+122.72%	[211]
468	Culture medium α-MEM	human PDL stem cells	Vibration	-	120	0.3	G	10	30 min. every 24 h.	Osteocalcin levels (OCN)	+154.54%	[211]
469	Culture medium DMEM low glucose	mesenchymal stem cells (MSCs)	Stretching	-	0.03	5	%	2	240 min/day	Collagen content	+26.22%	[67]
470	StemFit AK02N	iPSC	Vibration	-	100	0.02	mm	2	3000	Cells differentiation	−28.5%	[68]
471	StemFit AK02N	iPSC	Vibration	-	150	0.04	mm	2	3000	Cells differentiation	−22.85%	[68]
472	(DMEM)/Ham’s F12	human adipose-derived stem cells (hASCs)	Cyclic stretching	-	0.5	5%	%	2	864,000	Expression of *CYP1B1*	+68.18%	[69]
473	(DMEM)/Ham’s F12	human adipose-derived stem cells (hASCs)	Cyclic stretching	-	0.5	5%	%	2	864,000	Metabolic activity	−30.6%	[69]
474	Ham’s F–12 K	Chinese Hamster Ovary (CHO)-adherent cells	Vibration	-	10–500	<1	g	10	345,600	Cells proliferation	+79%	[56]
475	Freestyle CHO Expression	Chinese Hamster Ovary (CHO)- suspension cells	Vibration	-	10–500	<1	g	10	345,600	Cells proliferation	−13.0%	[56]
476	Freestyle CHO Expression	Chinese Hamster Ovary (CHO)- suspension cells	Vibration	-	30	0.7	g	10	345,600	Cells proliferation	+210%	[56]
477	RPMI	Е-cells	Vibration	-	30	0.7	g	10	345,600	Cells proliferation	+20.3%	[56]
478	DMEM	Primary fibrochondrocytes	Cyclic stretching	-	1	8	%	2	28,800	Expression of COL2A1	+110%	[59]
479	DMEM	Primary fibrochondrocytes	Cyclic stretching	-	1	8	%	2	28,800	Expression of SOX9	+200%	[59]
480	DMEM	Primary fibrochondrocytes	Cyclic stretching	-	1	8	%	2	28,800	Expression of ACAN	+215.78%	[59]
481	DMEM	Primary fibrochondrocytes	Cyclic stretching	-	1	8	%	2	28,800	Proportion of BrdU-positive cells	+46.6%	[59]
482	DMEM	Human osteoblast-like cells primary explant cultures	Vibration	-	60	30	µm	5	96 h	Confluency	+23.07%	[60]
483	DMEM	Human osteoblast-like cells primary explant cultures	Vibration	-	60	30	µm	5	96 h	Alkaline phosphatase activity	−29.82%	[60]
484	Culture medium α-MEM	GD25 cells	Vibration	-	12.5	0.5	G	5	12 days	Cells culture density	+50%	[61]
485	Culture medium α-MEM	GD25 cells	Vibration	-	12.5	0.5	G	5	15 days	Cells culture density	+60%	[61]
486	Culture medium α-MEM	GD25 cells	Vibration	-	12.5	0.5	G	5	18 days	Cells culture density	+83.67%	[61]
487	Culture medium α-MEM	GD25 cells	Vibration	-	12.5	0.5	G	5	21 day	Cells culture density	+73%	[61]
488	Culture medium α-MEM	GD25 cells	Vibration	-	12.5	0.5	G	5	24 days	Cells culture density	+45.8%	[61]
489	Culture medium α-MEM	GD25 cells	Vibration	-	12.5	0.5	G	5	15 days	Cell layer thickness	+46.15%	[61]
490	Culture medium DMEM	Human periodontal ligament stem cell (hPDLSC)	Shear stress	-	-	0.5	dyn/cm^2^	0.5	10,800	Expression of gene *IDO*	+80%	[62]
491	Culture medium DMEM	Human periodontal ligament stem cells (hPDLSC)	Shear stress	-	-	5	dyn/cm^2^	0.5	10,800	Expression of gene *IDO*	+140%	[62]
492	Culture medium DMEM	Human periodontal ligament stem cells (hPDLSC)	Shear stress	-	-	0.5	dyn/cm^2^	0.5	10,800	Expression of gene *COX2*	+83.3%	[62]
493	Culture medium DMEM	Human periodontal ligament stem cells (hPDLSC)	Shear stress	-	-	5	dyn/cm^2^	0.5	10,800	Expression of gene *COX2*	+266.6%	[62]
494	Culture medium DMEM	Human periodontal ligament stem cells (hPDLSC)	Shear stress	-	-	0.5	dyn/cm^2^	0.5	10,800	Activity of IDO	−18.18%	[62]
495	Culture medium DMEM	Human periodontal ligament stem cells (hPDLSC)	Shear stress	-	-	5	dyn/cm^2^	0.5	10,800	Activity of IDO	+106%	[62]
496	Culture medium DMEM	Human periodontal ligament stem cells (hPDLSC)	Shear stress	-	-	10	dyn/cm^2^	0.5	10,800	Activity of IDO	+78.78%	[62]
497	Culture medium DMEM	Human periodontal ligament stem cells (hPDLSC)	Shear stress	-	-	0.5	dyn/cm^2^	0.5	10,800	Kynurenine syntethis	+58.85%	[62]
498	Culture medium DMEM	Human periodontal ligament stem cells (hPDLSC)	Shear stress	-	-	5	dyn/cm^2^	0.5	10,800	Kynurenine syntethis	+170.5%	[62]
499	Culture medium DMEM	Human periodontal ligament stem cells (hPDLSC)	Shear stress	-	-	0.5	dyn/cm^2^	0.5	10,800	General concentration of TGF-β1 in medium	+17.3%	[62]
500	Culture medium DMEM	Human periodontal ligament stem cells (hPDLSC)	Shear stress	-	-	5	dyn/cm^2^	0.5	10,800	General concentration of TGF-β1 in medium	−9.6%	[62]
501	Culture medium DMEM	Human periodontal ligament stem cells (hPDLSC)	Shear stress	-	-	0.5	dyn/cm^2^	0.5	10,800	Concentration of active TGF-β1 in medium	+2.87%	[62]
502	Culture medium DMEM	Human periodontal ligament stem cells (hPDLSC)	Shear stress	-	-	5	dyn/cm^2^	0.5	10,800	Concentration of active TGF-β1 in medium	+125%	[62]
503	Culture medium DMEM	Human periodontal ligament stem cells (hPDLSC)	Shear stress	-	-	10	dyn/cm^2^	0.5	10,800	Concentration of active TGF-β1 in medium	+125%	[62]
504	Culture medium DMEM	Human periodontal ligament stem cells (hPDLSC)	Shear stress	-	-	5	dyn/cm^2^	0.5	10,800	Concentration of mature TGF-β1/β-ACTIN	−6.66%	[62]
505	Culture medium DMEM	Human periodontal ligament stem cells (hPDLSC)	Shear stress	-	-	10	dyn/cm^2^	0.5	10,800	Concentration of mature TGF-β1/β-ACTIN	−43.3%	[62]
506	Culture medium DMEM	Human periodontal ligament stem cells (hPDLSC)	Shear stress	-	-	0.5	dyn/cm^2^	0.5	10,800	Concentration of IFN-*γ* in lysate of cells	−51.21%	[62]
507	Culture medium DMEM	Human periodontal ligament stem cells (hPDLSC)	Shear stress	-	-	5	dyn/cm^2^	0.5	10,800	Concentration of IFN-*γ* in lysate of cells	−51%	[62]
508	Culture medium DMEM	Human periodontal ligament stem cells (hPDLSC)	Shear stress	-	-	10	dyn/cm^2^	0.5	10,800	Concentration of IFN-*γ* in lysate of cells	−65.85%	[62]
509	Culture medium DMEM	Chondrocytes from pig	Vibration	-	25	0.5	G	0.5	86,400	Type II collagen expression	+308.3%	[64]
510	Culture medium DMEM	Chondrocytes from pig	Vibration	-	25	0.5	G	0.5	86,400	Aggrecan expression	+225%	[64]
511	Culture medium DMEM	Chondrocytes from pig	Vibration	-	25	0.5	G	0.5	86,400	Type I collagen expression	+372.72%	[64]
512	Culture medium DMEM	Chondrocytes from pig	Vibration	-	25	0.5	G	0.5	86,400	Fibronectin expression	+206.25%	[64]
513	Culture medium α-MEM	Osteocytic MLO-Y4 cells	Vibration	-	45	0.5	g	0.5	259,200	Cells viability	+47.36%	[66]
514	Culture medium α-MEM	Osteocytic MLO-Y4 cells	Vibration	-	45	0.5	g	0.5	259,200	Proportion of apoptosis events	−47.36%	[66]
515	Culture medium α-MEM	Osteocytic MLO-Y4 cells	Vibration	-	45	0.5	g	0.5	259,200	Relative cell area	+52.17%	[66]
516	Culture medium α-MEM	Osteocytic MLO-Y4 cells	Vibration	-	45	0.5	g	0.5	259,200	Relative fluorescence intensity	+37%	[66]
517	Culture medium α-MEM	Osteocytic MLO-Y4 cells	Vibration	-	45	0.5	g	0.5	259,200	Cell anisotropy	+111%	[66]
518	Culture medium α-MEM	Osteocytic MLO-Y4 cells	Vibration	-	45	0.5	g	0.5	259,200	Expression of *Wnt3a*	+37.9%	[66]
519	Culture medium α-MEM	Osteocytic MLO-Y4 cells	Vibration	-	45	0.5	g	0.5	259,200	Expression of β-catenin	+52%	[66]
520	Culture medium α-MEM	Osteocytic MLO-Y4 cells	Vibration	-	45	0.5	g	0.5	259,200	Expression of Sost	−36.53%	[66]
521	Culture medium α-MEM	Osteocytic MLO-Y4 cells	Vibration	-	45	0.5	g	0.5	259,200	Expression of DKK1	−29.5%	[66]
522	Culture medium α-MEM	Osteocytic MLO-Y4 cells	Vibration	-	45	0.5	g	0.5	259,200	Expression of RANKL	−33.33%	[66]
523	Culture medium α-MEM	Osteocytic MLO-Y4 cells	Vibration	-	45	0.5	g	0.5	259,200	Expression of OPG	+62.2%	[66]
524	Culture medium α-MEM	Osteocytic MLO-Y4 cells	Vibration	-	45	0.5	g	0.5	259,200	Expression of PGE-2	+40%	[66]
525	Culture medium α-MEM	Osteocytic MLO-Y4 cells	Vibration	-	45	0.5	g	0.5	259,200	Expression of TNF- α	−31%	[66]
526	Culture medium α-MEM	Osteocytic MLO-Y4 cells	Vibration	-	45	0.5	g	0.5	259,200	Expression of RANKL	−20.5%	[66]
527	Culture medium α-MEM	Osteocytic MLO-Y4 cells	Vibration	-	45	0.5	g	0.5	259,200	Expression of OPG	+16.6%	[66]
528	Holtfreter’s saline	Newt *Triturus alpestri* notochord (tails)	US	-	1,000,000	8000	mW/cm^2^	-	300	Summ of cells with abnorman endoplasmatic reticulum	+7000%	[100]
529	TC culture mediun	Whole blood cells	US	-	2,250,000	30	mW	5	3600	Cell with chromosomal aberrations	+560	[111]
530	TC culture mediun	Whole blood cells	US	-	2,250,000	30	mW	5	7200	Cell with chromosomal aberrations	+620	[111]
531	TC culture mediun	Whole blood cells	US	-	2,250,000	30	mW	5	10,800	Cell with chromosomal aberrations	+360	[111]
532	TC culture mediun	Whole blood cells	US	-	2,250,000	30	mW	5	3600	Chromotid aberrations per 100 cells	+375	[111]
533	TC culture mediun	Whole blood cells	US	-	2,250,000	30	mW	5	7200	Chromotid aberrations per 100 cells	+800	[111]
534	TC culture mediun	Whole blood cells	US	-	2,250,000	30	mW	5	10,800	Chromotid aberrations per 100 cells	+375	[111]
535	TC culture mediun	Whole blood cells	US	-	2,250,000	30	mW	5	3600	Chromosome aberrations per 100 cells	+1100	[111]
536	TC culture mediun	Whole blood cells	US	-	2,250,000	30	mW	5	7200	Chromosome aberrations per 100 cells	+1800	[111]
537	TC culture mediun	Whole blood cells	US	-	2,250,000	30	mW	5	10,800	Chromosome aberrations per 100 cells	+700	[111]
538	TC culture mediun	Whole blood cells	US	-	2,250,000	30	mW	5	3600	Total aberrations	+520	[111]
539	TC culture mediun	Whole blood cells	US	-	2,250,000	30	mW	5	7200	Total aberrations	+1000	[111]
540	TC culture mediun	Whole blood cells	US	-	2,250,000	30	mW	5	10,800	Total aberrations	+440	[111]
541	Water	*Elodea* leaves cells with gas bodies sizes 10 × 14.7 μm	US	-	500,000	80	mW/cm^2^	0.5	100	Cell death	+100	[212]
542	Water	*Elodea* leaves cells with gas bodies sizes 10 × 14.7 μm	US	-	1,000,000	200	mW/cm^2^	0.5	100	Cell death	+100	[212]
543	Water	*Elodea* leaves cells with gas bodies sizes 10 × 14.7 μm=	US	-	400,000	140	mW/cm^2^	0.5	100	Cell death	+100	[212]
544	Water	*Elodea* leaves cells with gas bodies sizes 10 × 14.7 μm	US	-	1,500,000	400	mW/cm^2^	0.5	100	Cell death	+100	[212]
545	Water	*Elodea* leaves cells with gas bodies sizes 10 × 14.7 μm	US	-	200,000	500	mW/cm^2^	0.5	100	Cell death	+100	[212]
546	Water	*Elodea* leaves cells with gas bodies sizes 10 × 14.7 μm	US	-	250,000	450	mW/cm^2^	0.5	100	Cell death	+100	[212]
547	Water	*Elodea* leaves cells with gas bodies sizes 10 × 14.7 μm	US	-	500,0000	400	mW/cm^2^	0.5	100	Cell death	+100	[212]
548	Water	*Elodea* leaves cells with gas bodies sizes 10 × 14.7 μm	US	-	7,500,000	750	mW/cm^2^	0.5	100	Cell death	+100	[212]
549	Water	*Elodea* leaves cells with gas bodies sizes 10 × 14.7 μm	US	-	1,000,0000	1500	mW/cm^2^	0.5	100	Cell death	+100	[212]
550	Water	*Elodea* leaves cells with gas bodies sizes less than 5.3 × 7.4 μm	US	-	500,000	1100	mW/cm^2^	0.5	100	Cell death	+100	[212]
551	Water	*Elodea* leaves cells with gas bodies sizes less than 5.3 × 7.4 μm	US	-	1,000,000	1300	mW/cm^2^	0.5	100	Cell death	+100	[112]
552	Water	*Elodea* leaves cells with gas bodies sizes less than 5.3 × 7.4 μm	US	-	1,500,000	400	mW/cm^2^	0.5	100	Cell death	+100	[212]
553	Water	*Elodea* leaves cells with gas bodies sizes less than 5.3 × 7.4 μm	US	-	2,000,000	300	mW/cm^2^	0.5	100	Cell death	+100	[212]
554	Water	*Elodea* leaves cells with gas bodies sizes less than 5.3 × 7.4 μm	US	-	500,0000	200	mW/cm^2^	0.5	100	Cell death	+100	[212]
555	Water	*Elodea* leaves cells with gas bodies sizes less than 5.3 × 7.4 μm	US	-	1,000,000	900	mW/cm^2^	0.5	100	Cell death	+100	[212]
556	Isotonic saline	Healthy human latelets after incubation at 22 °C for 30 min	US	-	1,000,000	200	mW/cm^2^	1.5	300	Floculation during incubation	+10	[213]
557	Isotonic saline	Healthy human latelets after incubation at 22 °C for 30 min	US	-	1,000,000	600	mW/cm^2^	1.5	300	Floculation during incubation	+100	[213]
558	Isotonic saline	Red blood cells of healthy human	US	-	1,000,000	1000	mW/cm^2^	0.45	60	Hemolysis (percent)	+1900	[110]
559	Isotonic saline	Red blood cells of healthy human	US	-	1,000,000	2000	mW/cm^2^	0.45	60	Hemolysis (percent)	+2300	[110]
560	Isotonic saline	Red blood cells of healthy human	US	-	1,000,000	3000	mW/cm^2^	0.45	60	Hemolysis (percent)	+3200	[110]
561	Isotonic saline	Red blood cells of healthy human	US	-	1,000,000	4000	mW/cm^2^	0.45	60	Hemolysis (percent)	+1600	[110]
562	Isotonic saline	Red blood cells of healthy human	US	-	1,000,000	5000	mW/cm^2^	0.45	60	Hemolysis (percent)	+1400	[110]
563	Albunex solution	Red blood cells of healthy human	US	33 μL/mL	1,000,000	1000	mW/cm^2^	0.45	60	Hemolysis (percent)	+2600	[110]
564	Albunex solution	Red blood cells of healthy human	US	33 μL/mL	1,000,000	2000	mW/cm^2^	0.45	60	Hemolysis (percent)	+4500	[110]
565	Albunex solution	Red blood cells of healthy human	US	33 μL/mL	1,000,000	3000	mW/cm^2^	0.45	60	Hemolysis (percent)	+5600	[110]
566	Albunex solution	Red blood cells of healthy human	US	33 μL/mL	1,000,000	4000	mW/cm^2^	0.45	60	Hemolysis (percent)	+5100	[110]
567	Albunex solution	Red blood cells of healthy human	US	33 μL/mL	1,000,000	5000	mW/cm^2^	0.45	60	Hemolysis (percent)	+4800	[110]
568	photosensitive drug merocyanine 540 (MC 540)	HL-60 cell line	US	15 μg/mL	225,000	400	mW/cm^2^	5	30	Cell death	+49.5	[214]
569	Culture medium	*Bacillus subtilis* spores	US	-	67,000	50,000	mW	0.125	120	DNA releazing from spores	+800	[117]
570	Culture medium	*Bacillus subtilis* spores	US	-	67,000	50,000	mW	0.125	120	CFU counts growth from spores	−99	[117]
571	Culture medium	*Bacillus subtilis* spores	US	-	67,000	50,000	mW	0.125	120	DNA releazing from spores	+800	[117]
572	Culture medium	*Bacillus subtilis* spores	US	-	67,000	50,000	mW	0.125	120	DNA releazing from spores	+12800	[117]
573	Culture medium	*Bacillus subtilis* spores	US	-	67,000	50,000	mW	0.125	120	DNA releazing from spores	+25600	[117]
574	Culture medium	*Bacillus subtilis* spores	US	-	67,000	50,000	mW	0.125	120	DNA releazing from spores	+51200	[117]
575	Culture medium	*Bacillus subtilis* spores	US	-	67,000	50,000	mW	0.125	120	DNA releazing from spores	+204800	[117]
576	Culture medium	*Bacillus subtilis* spores	US	-	67,000	50,000	mW	0.125	120	DNA releazing from spores	+204800	[117]
577	RPMI1640	leukemic cells HL-60	US	-	750,000	103,700	mW/cm^−2^	15	30	DNA repair synthesis	+60	[109]
578	RPMI1640	leukemic cells HL-60	US	-	750,000	103,700	mW/cm^−2^	15	30	Cell viability	−45	[109]
579	RPMI1640	leukemic cells HL-60	US	-	750,000	103,700	mW/cm^−2^	15	30	Apoptose percantage	+200	[109]
580	RPMI1640	leukemic cells HL-60	US	-	750,000	103,700	mW/cm^−2^	15	30	Proliferation rate	−60	[109]
581	RPMI1640	leukemic cells K562	US	-	750,000	54,600	mW/cm^−2^	15	30	Apoptose percantage	+300	[109]
582	RPMI1640	leukemic cells U937	US	-	750,000	54,600	mW/cm^−2^	15	30	Apoptose percantage	+400	[109]
583	RPMI1640	leukemic cells M1/2	US	-	750,000	54,600	mW/cm^−2^	15	30	Apoptose percantage	+110	[109]
584	RPMI1640	leukemic cells HL-60	US	-	750,000	103,700	mW/cm^−2^	15	30	Late apoptose percantage	+300	[109]
585	RPMI1640	leukemic cells HL-60	US	-	750,000	22,400	mW/cm^−2^	15	30	Early apoptose percantage	+300	[109]
586	RPMI1640	leukemic cells HL-60	US	-	750,000	103,700	mW/cm^−2^	15	30	Early apoptose percantage	+400	[109]
587	RPMI1640	Healthy human platelet	US	-	22,000	1600	mW	5	10	Agregation rate	+15	[108]
588	RPMI1640	Healthy human platelet	US	-	22,000	1600	mW	5	30	Agregation rate	+35	[108]
589	RPMI1640	Healthy human platelet	US	-	22,000	1600	mW	5	60	Agregation rate	+55	[108]
590	RPMI1640	Healthy human platelet	US	-	22,000	1400	mW	5	15	Ca^2+^ concentration is cytoplasm	+100	[108]
591	RPMI1640	Healthy human platelet	US	-	22,000	1400	mW	5	30	Ca^2+^ concentration is cytoplasm	+180	[108]
592	RPMI1640	Healthy human platelet	US	-	22000	16,000	mW	5	60	Ca^2+^ concentration is cytoplasm	+900	[108]
593	RPMI-1640	K562 cells	US	-	1,800,000	220	mW/cm^−2^	2.5	1800	Early apoptic cells count	+410	[215]
594	RPMI-1640	K562 cells	US	-	1,800,000	220	mW/cm^−2^	2.5	7200	Early apoptic cells count	+740	[215]
595	RPMI-1640	K562 cells	US	-	1,800,000	220	mW/cm^−2^	2.5	18,000	Early apoptic cells count	+1450	[215]
596	RPMI-1640	K562 cells	US	-	1,800,000	220	mW/cm^−2^	2.5	7200	*bcl*-2 protein expression	+10000	[215]
597	RPMI-1640	K562 cells	US	-	1,800,000	220	mW/cm^−2^	2.5	7200	bax protein expression	+1000	[215]
598	YEPD medium	*E. coli strain DH5-alpha*	US	-	1,000,000	5200	mW/cm^−2^	0.002	30	Cell viability	−99	[118]
599	YEPD medium	*Saccharomyces cerevisiae*	US	-	1,000,000	5200	mW/cm^−2^	0.002	30	Cell viability	−99	[118]
600	Tryptic soy broth	*S. epidermidis*	US	-	70,000	3000	mW/cm^−2^	2	3600	Proliferation rate	+20	[216]
601	Tryptic soy broth	*S. epidermidis*	US	-	70,000	3000	mW/cm^−2^	2	7200	Proliferation rate	+20	[216]
602	Tryptic soy broth	*S. epidermidis*	US	-	70,000	3000	mW/cm^2^	2	10,800	Proliferation rate	+15	[216]
603	Healthy rabbits’ eyes	In vivo	US	-	880,000	190	mW/cm^2^	1	300	Sodium fluorescein concentration in the aqueous humor	+240	[99]
604	Healthy rabbits’ eyes	In vivo	US	-	880,000	340	mW/cm^2^	1	300	Sodium fluorescein concentration in the aqueous humor	+380	[99]
605	Healthy rabbits’ eyes	In vivo	US	-	880,000	560	mW/cm^2^	1	300	Sodium fluorescein concentration in the aqueous humor	+1060	[99]
606	Healthy rabbits’ eyes	In vivo	US	-	880,000	190	mW/cm^2^	1	300	Damaged cells	+179	[99]
607	Healthy rabbits’ eyes	In vivo	US	-	880,000	340	mW/cm^2^	1	300	Damaged cells	+174	[99]
608	Healthy rabbits’ eyes	In vivo	US	-	880,000	560	mW/cm^2^	1	300	Damaged cells	+171	[99]
609	MCDB-131 medium	Bovine aortic endothelial cells	US (continuous wave)	-	1,000,000	1200	mW/cm^2^	5	900	Cell proliferation after 48 h	+162	[114]
610	MCDB-131 medium	Bovine aortic endothelial cells	US (continuous wave)	-	3500,000	1200	mW/cm^2^	5	900	Cell proliferation after 48 h	+154	[114]
611	MCDB-131 medium	Bovine aortic endothelial cells	US (pulse wave)	-	1,000,000	1200	mW/cm^2^	5	900	Cell proliferation after 48 h	+135	[114]
612	MCDB-131 medium	Bovine aortic endothelial cells	US (pulse wave)	-	3500,000	1200	mW/cm^2^	5	900	Cell proliferation after 48 h	+123	[114]
613	50 mM CaCl_2_	*Pseudomonas putida* UWC1	US	-	40,000	240	mW/cm^−2^	0.5	10	Ultrasound DNA transfer	+100	[119]
614	50 mM CaCl_2_	*Escherichia coli* DH5α	US	-	40,000	240	mW/cm^−2^	0.5	10	Ultrasound DNA transfer	+100	[119]
615	50 mM CaCl_2_	*Pseudomonas fluorescens* SBW25	US	-	40,000	240	mW/cm^−2^	0.5	10	Ultrasound DNA transfer	+100	[119]
616	BG11 medium	*Microcystis aeruginosa* cyanobacteria	US	-	25,000	320	mW/cm^−2^	250	300	Proliferation	−90	[120]
617	BG11 medium	*Microcystis aeruginosa* cyanobacteria	US	-	25,000	320	mW/cm^−2^	250	300	Chlorophyll *a* concentration	−21	[120]
618	BG11 medium	*Microcystis aeruginosa* cyanobacteria	US	-	25,000	320	mW/cm^−2^	250	300	PC absorbance	−45	[120]
619	BG11 medium	*Microcystis aeruginosa* cyanobacteria	US	-	25,000	320	mW/cm^−2^	250	300	Extracellular microcystins	−17	[120]
620	BG11 medium	*Microcystis aeruginosa* cyanobacteria	US	-	25,000	320	mW/cm^−2^	250	300	Oxygen evolution rate	−40	[120]
621	F12 medium	Human Airway Smooth Muscle (HASM) cells	US	-	1,000,000	1000	mW/cm^−2^	0.8	300	Cell contractile moment	−40	[217]
622	F12 medium	Human Airway Smooth Muscle (HASM) cells	US	-	1,000,000	2000	mW/cm^−2^	0.8	300	Cell contractile moment	−65	[217]
